# Optimisation of anti-interleukin-6 therapy: Precision medicine through mathematical modelling

**DOI:** 10.3389/fimmu.2022.919489

**Published:** 2022-07-19

**Authors:** Jean-François Rossi, Hao-Chun Chiang, Zhao-Yang Lu, Kalle Levon, Frits van Rhee, Karan Kanhai, David C. Fajgenbaum, Bernard Klein

**Affiliations:** ^1^ Hématologie-Immunothérapie, Institut du Cancer Avignon-Provence, Sainte Catherine, Avignon, France; ^2^ Faculté de Médecine Montpellier, Université de Montpellier, Montpellier, France; ^3^ New York University (NYU) Tandon School of Engineering, Brooklyn, NY, United States; ^4^ Unité de Thérapie Cellulaire, CHU Montpellier Saint-Eloi, Montpellier, France; ^5^ Myeloma Center, University of Arkansas for Medical Sciences, Little Rock, AR, United States; ^6^ Medical Affairs, EUSA Pharma, Hemel Hempstead, United Kingdom; ^7^ Center for Cytokine Storm Treatment & Laboratory, Perelman School of Medicine, University of Pennsylvania, Philadelphia, PA, United States; ^8^ Division of Translational Medicine and Human Genetics, Perelman School of Medicine, University of Pennsylvania, Philadelphia, PA, United States

**Keywords:** mathematic model, cytokine storm, C-reactive protein, idiopathic multicentric castleman disease, COVID-19, siltuximab, tocilizumab, interleukin-6

## Abstract

**Background:**

Dysregulated interleukin (IL)-6 production can be characterised by the levels present, the kinetics of its rise and its inappropriate location. Rapid, excessive IL-6 production can exacerbate tissue damage in vital organs. In this situation, therapy with an anti-IL-6 or anti-IL-6 receptor (IL-6R) monoclonal antibody, if inappropriately dosed, may be insufficient to fully block IL-6 signalling and normalise the immune response.

**Methods:**

We analysed inhibition of C-reactive protein (CRP) – a biomarker for IL-6 activity – in patients with COVID-19 or idiopathic multicentric Castleman disease (iMCD) treated with tocilizumab (anti-IL-6R) or siltuximab (anti-IL-6), respectively. We used mathematical modelling to analyse how to optimise anti-IL-6 or anti-IL-6R blockade for the high levels of IL-6 observed in these diseases.

**Results:**

IL-6 signalling was insufficiently inhibited in patients with COVID-19 or iMCD treated with standard doses of anti-IL-6 therapy. Patients whose disease worsened throughout therapy had only partial inhibition of CRP production. Our model demonstrated that, in a scenario representative of iMCD with persistent high IL-6 production not controlled by a single dose of anti-IL-6 therapy, repeated administration more effectively inhibited IL-6 activity. In a situation with rapid, high, dysregulated IL-6 production, such as severe COVID-19 or a cytokine storm, repeated daily administration of an anti-IL-6/anti-IL-6R agent, or alternating daily doses of anti-IL-6 and anti-IL-6R therapies, could neutralise IL-6 activity.

**Conclusion:**

In clinical practice, IL-6 inhibition should be individualised based on pathophysiology to achieve full blockade of CRP production.

**Funding:**

EUSA Pharma funded medical writing assistance and provided access to the phase II clinical data of siltuximab for analysis.

## Introduction

**Figure 1:**
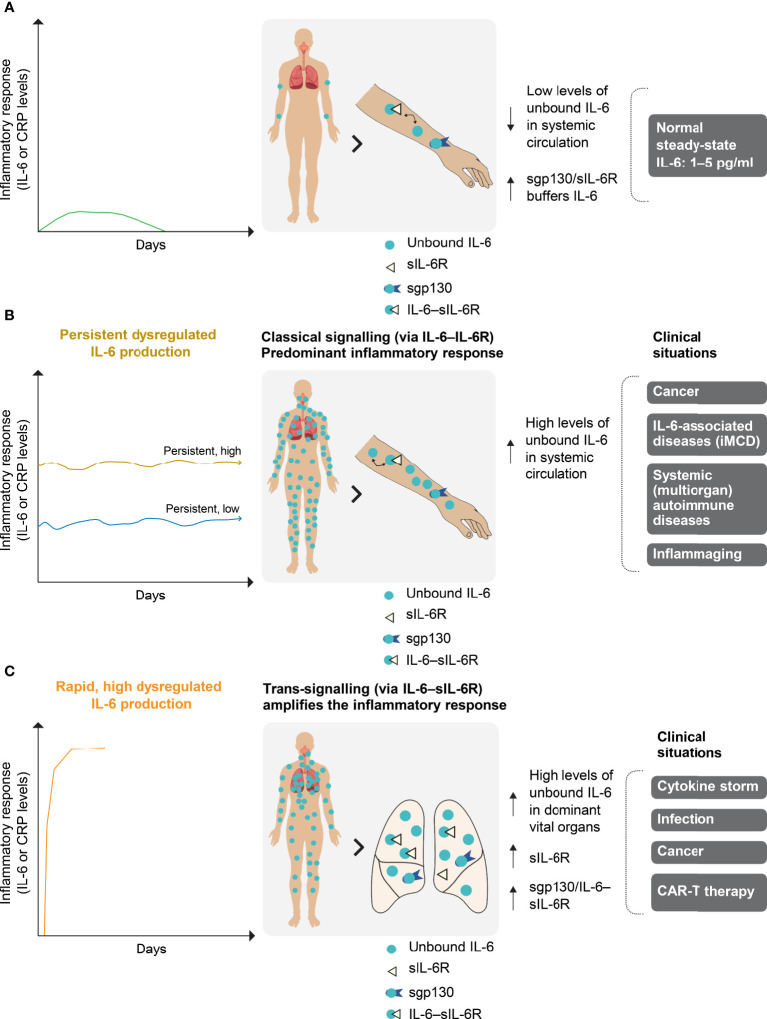
Normal **(A)** and dysregulated **(B, C)** IL-6 production. **(A)** In a normal transient IL-6 response, IL-6 is buffered by the sgp130–sIL-6R complex to ensure immune homeostasis. IL-6 is produced locally at the site of any injury and circulates systemically. The severity of IL-6 dysregulation is linked to the localisation of its production (i.e. in a vital or non-vital organ), as well as its dynamics, duration of increase and level. **(B)** Persistent dysregulated IL-6 production is seen in cancer, iMCD and systemic autoimmune diseases. The persistent high or low inflammatory response associated with these diseases occurs predominantly in the systemic compartment *via* classical IL-6 signalling. **(C)** Rapid and highly dysregulated levels of IL-6 induce pro-inflammatory trans-signalling that appears to be dominant in local vital organs (e.g. the lungs). This amplifies the inflammatory response and is a potent activator of endothelial activation, as observed during the cytokine storm that can occur in COVID-19, infections and cancers. iMCD, idiopathic multicentric Castleman disease; CAR-T, chimaeric antigen receptor T cells; CRP, C-reactive protein; gp130, glycoprotein 130; IL-6, interleukin-6; IL-6R, interleukin-6 receptor; s, soluble.

Interleukin (IL)-6 is a major pro-inflammatory cytokine that is involved in a variety of immune responses (1–3). Dysregulated inflammatory responses in certain infectious/inflammatory diseases and cancers (4, 5) are characterised by inappropriate levels of IL-6, the speed of their generation and their major site of production, such as a vital organ (**Figure 1**). The degree and rapidity of the inflammatory response are correlated with a patient’s prognosis, particularly in infection and cancer, and the response can eventually progress to a cytokine storm. In particular, COVID-19 severity and mortality are strongly associated with IL-6 levels (6, 7).

Plasma IL-6 levels are low in healthy individuals and mildly elevated in those experiencing a physiologically appropriate immune response (pg/ml range) (**Figure 1A**) (2, 8). However, high IL-6 levels are observed in idiopathic multicentric Castleman disease (iMCD), a rare hyperinflammatory disorder involving polyclonal lymphoproliferation (**Figure 1B**) (9). Dysregulated IL-6 production leads to levels 100–500 times higher than normal in patients experiencing hyperinflammation, as in severe acute respiratory distress syndrome (ARDS) associated with sepsis (6, 10, 11). IL-6 activity in ARDS is accentuated at the site of disease activity, and the local IL-6 concentration in bronchoalveolar lavage fluid can be 10-fold higher than in the circulation (**Figure 1C**) (12). Both plasma and bronchoalveolar lavage fluid IL-6 concentrations are high in patients with ARDS associated with COVID-19 (11, 13).

The first anti-IL-6 monoclonal antibody (mAb) treatments were developed in 1991, and studies demonstrated that hepatic C-reactive protein (CRP) production could be fully controlled by IL-6 in humans (14). In some patients with multiple myeloma treated with anti-IL-6 therapy, serum CRP was completely inhibited; however, levels increased in the 3–4 days following treatment cessation (14). These initial studies, using mathematical modelling of the inhibition of IL-6 signalling by the mAb, found it was possible to predict the ability of an anti-IL-6 mAb to block plasma IL-6 bioactivity and showed that IL-6 inhibition depended on the extent of whole-body IL-6 production (15).

IL-6 targeting is an effective therapeutic approach for patients with iMCD (characterised by persistent dysregulated IL-6 production; **Figure 1B**). In a phase II study of 79 patients with iMCD, durable symptomatic and tumour responses were seen in 34% of patients treated with the anti-IL-6 therapy siltuximab, compared with no patients in the placebo arm (9). Although there was a trend towards a higher response rate among patients with high IL-6 levels, others with low or normal values also responded to siltuximab, whereas some patients with high levels did not (9). For some patients with iMCD, therefore, full IL-6 suppression is not achieved or maintained with current approaches to IL-6 blockade.

IL-6 targeting is also an effective therapy for patients experiencing rapid, high, dysregulated IL-6 production (**Figure 1C**). A prospective meta-analysis of clinical trials of patients hospitalised for COVID-19 showed that administration of IL-6 antagonists, compared with usual care or placebo, was associated with lower 28-day all-cause mortality (16). However, most trials assessing anti-IL-6 or anti-IL-6 receptor (IL-6R) agents have either failed to monitor CRP levels or to achieve complete CRP inhibition (17).

We postulate that, in patients in whom the IL-6 concentration is likely higher at the site of inflammation than in plasma (**Figure 1C**), full blockade of plasma IL-6 activity, as evaluated by complete CRP inhibition in the liver, is the minimum requirement for clinical efficacy. To better define the use of IL-6 therapies, we developed an algorithm to mathematically model inhibition of IL-6 activity in the presence of siltuximab (an anti-IL-6 therapy), tocilizumab (an anti-IL-6R therapy) or both in two scenarios: first, representative of iMCD, with persistently high IL-6 production; and second, representative of severe COVID-19, with a cytokine storm and massive IL-6 production.

## Materials and methods

Using an exponential function, we showed a correlation between plasma CRP and IL-6 levels in various diseases (*r^2 =^* 0.9966, p*=*2.71×10^–7^, **Supplementary Figure 1**), confirming basic research showing that IL-6 is mandatory for CRP production (18, 19) and that CRP can be used as a proxy measure for IL-6 bioactivity.

### CRP inhibition in patients with iMCD treated with siltuximab

We performed an exploratory *post hoc* analysis of data from a phase II randomised, double-blind, placebo-controlled trial of siltuximab in patients with iMCD (9). In the phase II study, CRP levels were measured in patients receiving siltuximab (11 mg/kg every 3 weeks; n=53) or placebo (n=26) weekly for the first 3 weeks, then once every 3 weeks for a total period of 48 weeks (9). Patients received best supportive care, which included management of effusions, use of antipyretic, antipruritic, antihistamine and pain drugs, management of infections, transfusions, and standard management of infusion-related reactions as specified in institutional guidelines. Use of erythropoietin-stimulating agents, anti-tumour treatments, biological treatments, or an increase from baseline or a new course of corticosteroids were not allowed (9).

We classified patients as having low or high CRP levels at baseline using a threshold of 40 mg/l (corresponding to approximately 40 pg/ml of IL-6; selected based on our clinical experience and the mean [standard deviation] baseline CRP level in the siltuximab arm of 43.18 [53.63] mg/l). To evaluate CRP inhibition, one-way repeated measures of analysis of variance followed by Tukey’s *post hoc* correction were performed using data from patients with iMCD treated with siltuximab (high and low baseline CRP groups) for CRP measured on days 1, 8 and 15 (all CRP measurements taken in the first treatment cycle of the study). A p-value of ≤ 0.05 was considered significant.

### CRP inhibition in patients with COVID-19 treated with tocilizumab

In contrast to iMCD, where siltuximab is the only drug tested in a randomised trial, there are many reports in the literature evaluating therapies in patients with COVID-19. In June 2022, we searched ClinicalTrials.gov and the published literature (PubMed) to identify studies evaluating the efficacy, or assessing the potential role, of anti-IL-6 therapy in patients with severe COVID-19. We found 53 studies with tocilizumab, two with siltuximab and nine with sarilumab, plus a prospective meta-analysis of 27 randomised trials that included 10,930 patients hospitalised for COVID-19. The meta-analysis concluded that administration of IL-6 antagonists, compared with usual care or placebo, was associated with significantly lower 28-day all-cause mortality. Surprisingly, only one study, by Luo et al. (2020), reported the dynamics of CRP reduction for individual patients throughout therapy (20).

We analysed the previously published data of Luo et al. (20) showing CRP inhibition in patients with COVID-19 treated with tocilizumab. We plotted data for patients with multiple CRP data points against a hypothetical curve showing optimal CRP inhibition. This curve was based on the half-life of CRP and showed CRP levels that would reflect a complete block of IL-6.

### Mathematical modelling of IL-6 Bioactivity in two clinical scenarios: iMCD and severe COVID-19 with a cytokine storm

We developed a model by expanding and modifying the basic structure of a previously developed model (10, 15) to accommodate observations from the experimental literature and our own experimental data (**Supplementary Table 1**) (10, 11, 15, 20). The model was built using the SimBiology package in MATLAB 2019b. It comprised 17 biological elements (e.g. IL-6, IL-6R, gp130) with 20 biological reactions to represent their time-dependent concentrations (**Supplementary Table 1**).

**Figure 2:**
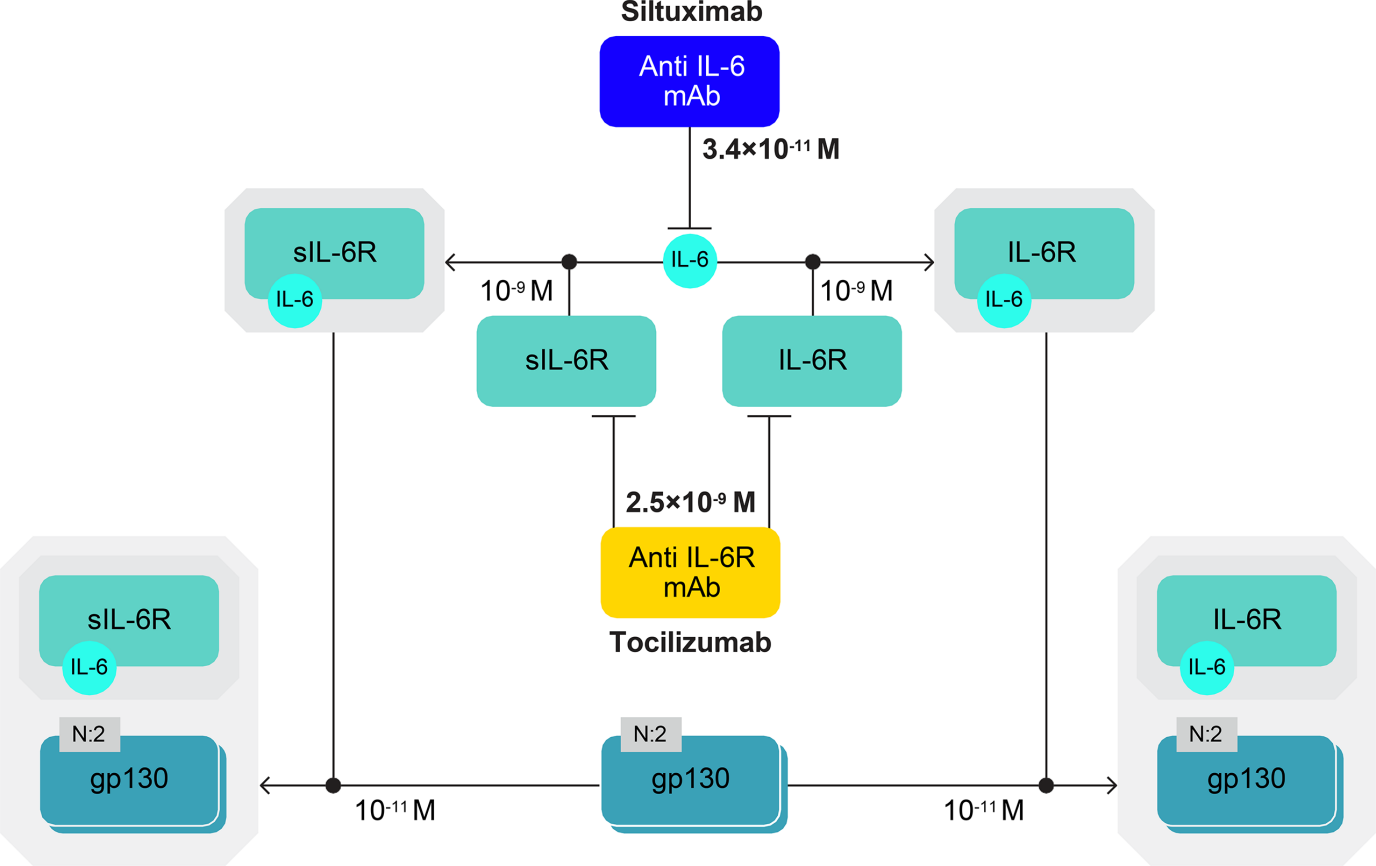
IL-6–(IL-6R/sIL-6R)–gp130 complexes. IL-6 signal transduction is initiated when IL-6 binds to its membrane-bound receptor (IL-6R) or plasma-soluble receptor (sIL-6R) and IL-6–IL-6R and IL-6–sIL-6R complexes bind to a membrane glycoprotein transducer (gp130). Affinity constants are shown for the IL-6 complex molecules and the anti-IL-6 (siltuximab) and anti-IL-6R (tocilizumab) mAbs, which are approved for use in humans. Siltuximab has a higher binding affinity for IL-6 than tocilizumab has for IL-6R. The diagram has been produced following the Systems Biology Graphical Notation. N:2 indicates a multimer with two subunits (e.g. gp130 dimer). gp130, glycoprotein 130; IL-6, interleukin-6; IL-6R, interleukin-6 receptor; mAb, monoclonal antibody; s, soluble.

IL-6 binds to its membrane-bound receptor (IL-6R) or plasma-soluble receptor (sIL-6R). Both IL-6–IL-6R and IL-6–sIL-6R complexes bind to a membrane glycoprotein transducer (gp130), initiating signal transduction (**Figure 2**) (1–4). Our algorithm was developed to take into account these components of IL-6 signalling to model the formation of IL-6–(IL-6R/sIL-6R)–gp130 complexes and then of CRP production, which serves as a proxy for IL-6 bioactivity. The algorithm included reactions in a general plasma compartment and a local immobile compartment (lymph nodes for iMCD and bronchoalveolar fluid [BAF] for COVID-19).

The model was fitted to experimental data for IL-6, glycoprotein (gp)130 and CRP simultaneously with a proportional error model. This was chosen based on the large order of magnitude variation in the data across the experiments.


$$y_{exp}=y_{sim}+b\left|ysim\right|\text{error}$$

where y_exp_ is biological species, y is experimental response, y_sim_ is biological species y simulation value and b is the proportional error parameter. IL-6R and gp130 in the solution form were tagged ‘s’; IL-6 and mAb could only be in solution form.

The reaction system was divided into a general plasma compartment and an immobile compartment present at the cellular membrane. One of the simplest approaches to model these biological reactions is to use a ‘curve fitting’ to construct a curve (mathematical model) that has the best fit to series of known data. The algorithm of stiff ode15s solver was used with settings as shown in **Supplementary Table 1**. A tolerance of 1e–5 was chosen for termination of the data-fitting procedure for the step change in estimated parameters and the function value, and 1e–6 for the first-order optimality. This fitting was performed in an iterative fashion as the model contained numerous parameters to allow efficient simultaneous estimation.

A molar equivalent-order kinetic equation was used for the binding of receptor and mAb in the solution. The kinetic equation of surface-immobilised ligand and protein was used for interactions on the membrane. Catabolism and appeared clearance were also described by mass/molar action. The degradation rate or clearance rate for non-structural proteins was estimated using the half-life (t_1/2_). For example, if the value for the plasma half-life cycle of sIL-6R was 2 hours, κ would be 0.277. This estimation of degradation rates was found to be acceptable for our model after fitting, and aided in reducing the degrees of freedom in the fitting process. In addition, degradation rates for all biological species on the membrane were estimated to be zero based on previous studies (10, 15), showing complete recovery in the hepatocyte compartment (e.g. gp130). In plasma, different cases of steady-state IL-6 and sIL-6R levels were simulated with a constant generation rate. The steady-state level of sIL-6R is 75 ng/ml and IL-6 levels are in the range of 1–10,000 pg/ml. The program performed the simulation for 15 days (360 hours) and the observed biological species had to reach a stable level before applying treatment. A simulation can be reproduced using this software and the above settings.

The ‘Sensitivity Analysis’ task of the SimBiology package was used to estimate parameter sensitivities for IL-6, IL-6R and CRP production, as shown in **Supplementary Figure 5**. This task uses the complex-step derivative approximation, which is often used for metabolic systems. Full dedimensionalisation of the analysis was used to permit comparisons of parameters with widely different orders of magnitude. The calculation for complex-step derivative sensitivities can be represented by the following equations for each parameter:


$$\begin{array}{c} f^{\prime }=\frac{df}{d\left(k_{\text{reaction}}\right)}\\ f\left(k_{\text{reaction}},t\right)=\text{species}\times \text{per cell}\\ Sensitivity=\int_{0}^{t_{final}}f^{\prime }\left(t\right)dt \end{array}$$

where *f* = is the first derivative of the simulation function with respect to a given reaction rate *k*_reaction_ and *t* is time.

The iMCD scenario was modelled with persistent high IL-6 secretion, at 1 ng/ml – the median concentration reported for patients with iMCD (9). The severe COVID-19 scenario (with a cytokine storm and massive IL-6 production) used 3 ng/ml IL-6 in BAF – the median concentration reported for patients with severe COVID-19 (13).

The diffusion of mAbs into bronchoalveolar lavage fluid is unknown but is expected to be low (~0.1–0.3%) according to the diffusion data for an anti-respiratory syncytial virus mAb into bronchoalveolar lavage fluid in animal models (21). In contrast, the diffusion of mAbs into lymph nodes occurs more freely through the sinusoidal clefts, which allows mAb movement and biodistribution, based on rituximab-mediated B-cell depletion in lymph nodes, and was estimated to be 8.46% of the plasma concentration (22). For this reason, we modelled primarily with local mAb concentrations at 10% of those in plasma. To account for the uncertainty (particularly in the scenario of COVID-19), we also modelled various situations using concentrations from 1% to 100% of those in plasma, and present these results as **Supplementary Data**.

### Modelling inhibition of IL-6/CRP in the presence of siltuximab, tocilizumab or their combination

Using the mathematical model described above, we modelled the ability of tocilizumab and siltuximab, or a combination of both, to inhibit IL-6 bioactivity (i.e. formation of IL-6/IL-6R–gp130 and IL-6/sIL-6R–gp130 complexes). The inhibition rate (%) versus pre-treatment levels was defined as the ratio of gp130 complexes – IL-6/IL-6R–gp130 and IL-6/sIL-6R–gp130 – after applying mAb treatments to reach their stable level. We also computed CRP levels; we considered a 1-day delay for induction of CRP production by IL-6. Results show the concentration of CRP in the circulating compartment. Daily IL-6 production was assumed to be constant throughout treatments.

Doses of siltuximab and tocilizumab used in the model were selected based on clinical experience. Siltuximab 700 mg was used, as this represents the median dose of siltuximab for a person weighing 75–80 kg. A higher dose of tocilizumab (800 mg) was used because some patients with COVID-19 have been administered two injections of 400 mg each (**Supplementary Table 2**). In the modelled scenarios, siltuximab (700 mg) or tocilizumab (800 mg) was either injected once at day 0 (D0) or given as repeated daily injections (D0, D1, D2, D3, —, Dx). A further scenario modelled alternating daily injections of siltuximab and tocilizumab (siltuximab: D0, D2, —, Dx; tocilizumab: D1, D3, —, Dx+1).

Given previous estimates indicating weak diffusion of plasma mAb into lymph nodes and interstitial lung tissue (8.46% and 14.9% of that in plasma, respectively) (21, 22) and the established pharmacokinetics/pharmacodynamics of siltuximab (23) and tocilizumab (24), we assumed the mAb concentration in lymph nodes (iMCD scenario) or BAF (COVID-19 scenario) to be 10% of that in plasma. Because of uncertainty around mAb biodistribution, particularly into BAF (unknown but expected to be low: ~0.1–0.3% of that in plasma; see **Supplementary Methods**), we also include results with mAb penetration into lymph nodes and BAF of 100% and 1% of the concentration in plasma as **Supplementary Data**.

## Results

### In patients with iMCD, CRP inhibition in response to siltuximab treatment differed according to baseline CRP level

**Figure 3:**
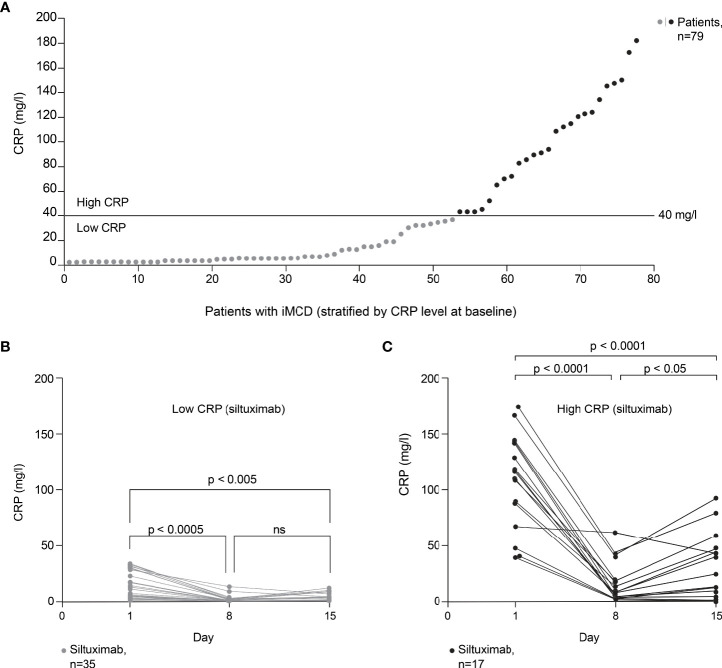
Effect of baseline CRP level on response to siltuximab in patients with iMCD. Exploratory *post hoc* analysis of patients with iMCD from study NCT01024036 (siltuximab, n=53; placebo, n=26) (9). **(A)** Patients (n=79) classified as having low or high baseline CRP levels using a threshold of 40 mg/l (corresponding to approximately 40 pg/ml of IL-6). **(B)** In patients with low baseline CRP levels (≤40 mg/l) who were treated with siltuximab (n=35), CRP levels were significantly reduced versus baseline on day 8 (n=26; p*=*0.0003) and day 15 (n=22; p*=*0.0043). **(C)** In patients with high baseline CRP levels (>40 mg/l) who were treated with siltuximab (n=17), CRP levels were significantly reduced versus baseline on day 8 (n=17; p *<* 0.0001) and day 15 (n=15; p *<* 0.0001). A significant increase in CRP levels from day 8 to day 15 was observed in this patient group (p*=*0.0120). One-way analysis of variance and Tukey’s multiple comparisons tests were used for all analyses. CRP, C-reactive protein; IL-6, interleukin-6; iMCD, idiopathic multicentric Castleman disease; ns, not significant.

Substantial variability was observed in the baseline CRP levels of patients with iMCD who participated in the phase II trial of siltuximab (9). Across the 79 patients with iMCD (siltuximab, n=53; placebo, n=26), baseline CRP levels were 0.39–181 mg/l (**Figure 3A**). To assess the impact of baseline CRP levels on response to siltuximab, patients were divided into low and high CRP groups based on a threshold CRP value of 40 mg/l (corresponding to approximately 40 pg/ml of IL-6 (25)).

In patients with low baseline CRP levels (≤40 mg/l) who were treated with siltuximab (n=35), CRP levels were significantly reduced versus baseline on day 8 (n=26; p=0.0003) and day 15 (n=22; p=0.0043) post dosing (**Figure 3B**). In patients with high baseline CRP levels (>40 mg/l) who were treated with siltuximab (n=17), CRP levels were significantly reduced versus baseline on day 8 (n=17; p < 0.0001) and day 15 (n=15; p < 0.0001) post dosing (**Figure 3C**). However, a significant increase in CRP levels from day 8 to day 15 was observed in this group (p=0.0120); this increase was not observed in patients with low baseline CRP levels (p=0.243). Moreover, for some patients with high baseline CRP, CRP levels remained above 40 mg/l after siltuximab treatment. For these patients, a single dose of siltuximab did not efficiently inhibit IL-6 activity.

### CRP inhibition with tocilizumab in patients with COVID-19

**Figure 4:**
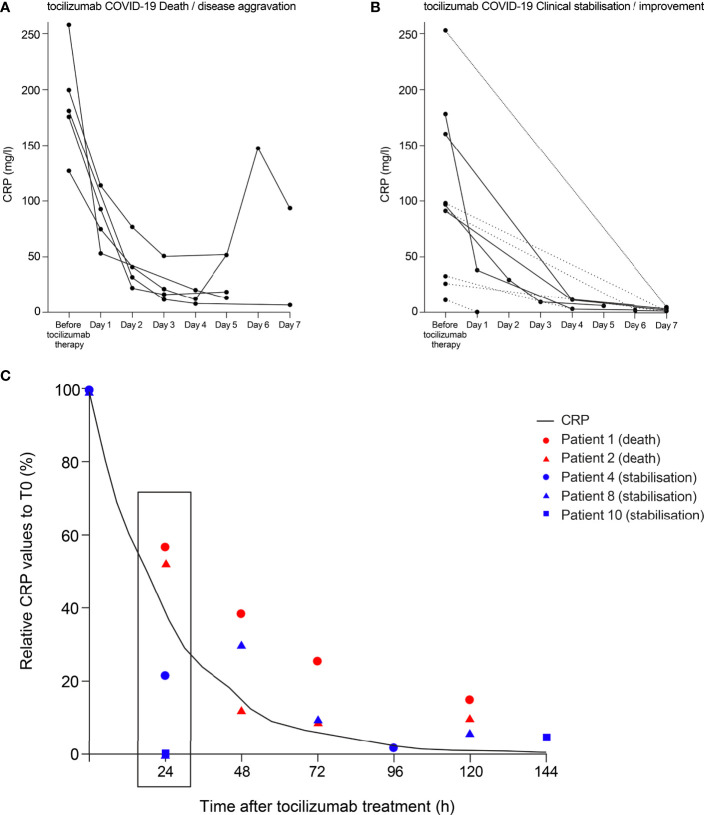
Theoretical curve for CRP inhibition to achieve complete IL-6 blockade compared with CRP levels in patients with COVID-19 treated with tocilizumab. CRP data points from patients with COVID-19 treated with tocilizumab in the study of Luo and colleagues (1) are shown. Individual data points are plotted for CRP values from **(A)** patients who died or experienced disease aggravation following tocilizumab treatment and **(B)** patients who experienced clinical stabilisation or improvement following tocilizumab treatment. CRP levels were not measured at every time point for every patient. Two patients died on day 6 and one patient died on day 7; the last CRP measurements for these patients were at day 5. For patients with fewer than three CRP measurements, data points are joined with a dotted line. **(C)** Relative CRP values to the initial concentration are depicted for patients who had several CRP measurements. Patients who died following tocilizumab treatment are shown in red and those whose condition stabilised are shown in blue. The curve plotted shows the percentage of ideal CRP reduction that reflects a complete block of IL-6, taking into account its half-life of 18 hours. CRP, C-reactive protein; IL-6, interleukin-6; T0, time 0.

To our knowledge, only one study (20) has reported the dynamics of CRP reduction for individual patients with severe COVID-19 treated with anti-IL-6 therapy. To evaluate the completeness of IL-6 activity inhibition in patients with severe COVID-19 treated with the anti-IL-6R therapy tocilizumab in the study by Luo et al. (20), we examined CRP levels in patients who died or had disease aggravation (n=5) and those with clinical stabilisation or improvement (n=10) following tocilizumab administration. In general, incomplete CRP reduction was observed in patients who died or experienced disease aggravation following treatment with tocilizumab (**Figure 4A**). The three patients who died on, or before, day 7 had CRP levels of 52, 18 and 13 mg/l at the last reported measurement. One patient who survived beyond day 7 but experienced disease aggravation had a CRP level of 94 mg/l on day 7. Another patient whose CRP levels decreased to 6 mg/l at day 7 also experienced disease aggravation despite the reduction in CRP. In patients with clinical stabilisation or improvement following treatment with tocilizumab, CRP levels were more effectively suppressed (**Figure 4B**), with all CRP levels ≤5 mg/l at last reported measurement (median 2.3, range 0.5–5).

To further evaluate this trend, we selected patients with several CRP measurements to assess the dynamics of the relative reduction (%) in CRP serum values. We compared the reduction in CRP to a hypothetical curve of CRP reduction required for complete block of IL-6 (taking into account the 18-hour half-life of CRP). The two patients who died had early CRP reduction rates (at 24 hours post tocilizumab treatment) above the theoretical curve representing a complete block of IL-6; conversely, at 24 hours post tocilizumab treatment, the three patients who stabilised had CRP reduction rates below the theoretical curve (**Figure 4C**).

### Inhibition of IL-6 activity by Anti-IL-6 and Anti-IL-6R mAbs as monotherapy, intensified or combined therapy

We modelled a situation representative of iMCD, with persistent high IL-6 secretion, at 1 ng/ml IL-6, the median concentration reported for patients with iMCD (9). We also modelled a situation representative of severe COVID-19, with a cytokine storm and massive IL-6 production (3 ng/ml IL-6 in BAF – the median concentration reported for patients with severe COVID-19 (13)). Assuming a mAb concentration in lymph nodes or BAF of 10% that in plasma (see *Materials and Methods*), we generated theoretical curves showing the efficacy of siltuximab, tocilizumab or a combination of the two mAbs in reducing the formation of gp130 complexes (a surrogate measure for IL-6 bioactivity) and plasma CRP levels produced in the liver.

**Figure 5:**
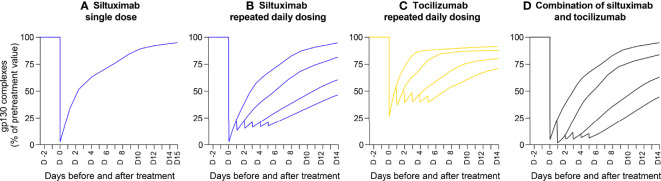
Mathematical modelling demonstrating inhibition of IL-6 activity (measured by gp130 complex formation) by an anti-IL-6 mAb, anti-IL-6R mAb and their combination. Outputs of a mathematical model of a situation representative of idiopathic multicentric Castleman disease (with persistent high IL-6 secretion, at 1 ng/ml). Penetration of mAb into the lymph nodes was modelled at 10% of the concentration in plasma. Theoretical curves show the efficacy of siltuximab, tocilizumab and a combination of the two mAbs in reducing the formation of gp130 complexes (a surrogate measure for IL-6 bioactivity). Panels show the inhibition of gp130 complex formation in response to: **(A)** a single injection of siltuximab, **(B)** repeated daily injections of siltuximab, **(C)** repeated daily injections of tocilizumab and **(D)** alternating daily injections of siltuximab and tocilizumab. gp130, glycoprotein 130; IL-6, interleukin-6; IL-6R, interleukin-6 receptor; mAb, monoclonal antibody.

Applying the model to the scenario representative of iMCD (with persistent high IL-6 secretion at 1 ng/ml and mAb penetration into lymph nodes at 10% of the plasma concentration), a single administration of siltuximab suppressed IL-6 bioactivity by 80%. Similar to the clinical data in patients with iMCD with high baseline CRP levels (**Figure 3C**), IL-6 bioactivity increased over the next 14 days to 90% of pre-treatment levels (**Figure 5A**). Repeated injections of siltuximab every 24 hours produced more sustained inhibition than a single injection, with IL-6 bioactivity plateauing at 82% inhibition (**Figure 5B**). Tocilizumab inhibited IL-6 bioactivity to a lesser extent than siltuximab; repeated daily injections of tocilizumab caused IL-6 bioactivity to plateau at 60% inhibition (**Figure 5C**). Theoretical administration of alternating daily injections of siltuximab and tocilizumab had a marginally greater effect in suppressing IL-6 bioactivity than siltuximab monotherapy (**Figure 5D**).

**Figure 6:**
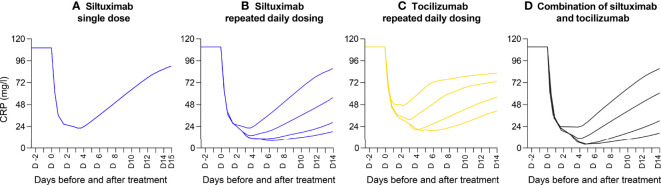
Mathematical modelling demonstrating the inhibition of IL-6 activity (measured by CRP formation) by an anti-IL-6 mAb, an anti-IL-6R mAb and their combination. Outputs of a mathematical model of a situation representative of idiopathic multicentric Castleman disease (with persistent high IL-6 secretion, at 1 ng/ml). Penetration of mAb into the lymph nodes was modelled at 10% of the concentration in plasma. Theoretical curves show the efficacy of siltuximab, tocilizumab and a combination of the two mAbs in reducing the formation of CRP in the circulation (a surrogate measure for IL-6 bioactivity). Panels show the inhibition of CRP formation in response to: **(A)** a single injection of siltuximab, **(B)** repeated daily injections of siltuximab, **(C)** repeated daily injections of tocilizumab and **(D)** alternating daily injections of siltuximab and tocilizumab. CRP, C-reactive protein; IL-6, interleukin-6; IL-6R, interleukin-6 receptor; mAb, monoclonal antibody.

Because CRP is measured in clinical practice and used as a surrogate measure for IL-6 bioactivity when administering anti-IL-6 therapies, we repeated the simulations described above to determine the inhibition of CRP in response to siltuximab, tocilizumab or both (**Figure 6**). CRP inhibition reflected the results observed for inhibition of gp130 complex formation.

In a situation representative of severe COVID-19 (3 ng/ml IL-6 in BAF; mAb penetration into BAF 10% of that in plasma), a single injection of siltuximab inhibited IL-6 bioactivity by 90%, with an immediate resumption of IL-6 bioactivity within 24 hours (**Supplementary Figure 2A**). Due to the continuous daily production of IL-6 (which was factored into the model), repeated daily dosing was required to maintain IL-6 bioactivity inhibition. Repeated injections of siltuximab every 24 hours caused IL-6 bioactivity to plateau with 65% inhibition (**Supplementary Figure 2A** and **Supplementary Table 3**). A single tocilizumab injection inhibited IL-6 bioactivity by 52%, with immediate restoration of IL-6 bioactivity within 24 hours. Repeated daily injections of tocilizumab caused IL-6 bioactivity to plateau with 38% inhibition (**Supplementary Figure 2A** and **Supplementary Table 3**). Alternating daily injections of siltuximab and tocilizumab produced maximal inhibition of IL-6 bioactivity, with a brief inhibition of 94% followed by a sustained plateau of inhibition at 85% (**Supplementary Figure 2A** and **Supplementary Table 3**). Similar results were obtained when CRP was used as a biomarker for IL-6 bioactivity (**Supplementary Figure 2B** and **Supplementary Table 3**).

To account for uncertainty around the diffusion of mAbs into BAF and lymph nodes, particularly when inflammation is present, we replicated the simulations described above with various diffusion abilities of the mAbs. In both iMCD and COVID-19, increasing the local mAb concentration from 10% to 100% increased inhibition of IL-6 bioactivity, with a sustained full blockade of IL-6 bioactivity with combination treatment, even in the scenario of severe COVID-19 with a cytokine storm (**Supplementary Figure 3**). In the iMCD scenario with mAb concentrations in lymph nodes at 1% of those in plasma, repeated daily administration of siltuximab caused IL-6 bioactivity to plateau at 35% inhibition, with a similar plateau observed for alternating daily injections of siltuximab and tocilizumab (**Supplementary Figure 4**). Repeated daily administration of tocilizumab caused IL-6 bioactivity to plateau at 20% inhibition. In the COVID-19 scenario with mAb concentrations at 1% of those in plasma, IL-6 bioactivity was minimally inhibited even with repeated daily injections of anti-IL-6 or anti-IL-6R mAb, either alone or in combination (**Supplementary Figure 4**). The plateaus of gp130 inhibition with repeated administration of siltuximab, tocilizumab or both mAbs at various diffusion abilities of the mAbs (1%, 10%, 20%, 40% and 100% of that in plasma) are shown in **Supplementary Table 3**.

## Discussion

In patients with a dysregulated inflammatory response – characterised by the rapid and massive production of IL-6, such as during a cytokine storm in patients with infection, iMCD or cancer – it is critical to bring the inflammatory response under control. Using evidence from clinical trials, we have shown that incomplete IL-6 inhibition in diseases involving rapid, high, dysregulated IL-6 production or persistent high IL-6 production may be associated with lack of disease control.

Our analysis shows heterogeneous baseline levels of CRP in patients with iMCD, and that patients with high baseline levels have significantly increased CRP levels within a week of initial inhibition observed with siltuximab therapy. Thus, the strategy for blocking IL-6 should be individualised based on dynamic monitoring of CRP levels, with treatment intensification warranted in patients experiencing a rebound in CRP levels to maintain IL-6 normalisation. Our algorithm suggests that repeated administration of anti-IL-6 and/or anti-IL-6R therapy may be necessary to optimise treatment in some scenarios. However, using a combination of siltuximab and tocilizumab requires careful consideration of the safety profiles of the two therapies and the ability of critically ill patients to tolerate the combination. Whether it is clinically feasible to combine therapies remains to be seen – an evaluation of therapy intensification (through repeated administration or elevated dosing) may be a more appropriate starting point for prospective clinical trials in iMCD.

We also present evidence for incomplete IL-6 inhibition in patients with severe COVID-19 treated with tocilizumab. This suggests that a single dose of tocilizumab is insufficient to suppress the inflammatory response, potentially limiting its ability to improve the condition of critically ill patients. Our model shows that, in a situation with rapid, high, dysregulated IL-6 production – such as severe COVID-19 or a cytokine storm – an initial dose of either an anti-IL-6 or anti-IL-6R mAb inhibits IL-6 bioactivity. However, this activity returns within 24 hours due to continued IL-6 production (**Supplementary Figure 2**). We demonstrate that repeated daily administration of the mAb is necessary to maintain a plateau of inhibition of IL-6 bioactivity. In the modelled scenario, an anti-IL-6 antibody (siltuximab) achieved more effective blockade of IL-6 signalling than an anti-IL-6R mAb (tocilizumab), likely due to siltuximab’s 100-fold affinity for IL-6 compared with tocilizumab’s affinity for IL-6R (**Figure 2**). This could, in part, also be due to differences in their modes of action. Our results imply that 100 μg/ml tocilizumab (the concentration in plasma following the usual dose in humans (24)) can block IL-6 concentrations of no more than 1 ng/ml. Importantly, anti-IL-6 and anti-IL-6R mAbs have been found to reduce systemic CRP levels to less than 4 and 1 mg/l (23, 26), respectively, which does not indicate full inhibition of IL-6 bioactivity. We believe that repeated administration of mAbs is needed as IL-6 is produced daily (10, 11) and levels recover following the first dose.

Moreover, our model shows that the use of siltuximab and tocilizumab on alternate days increases the probability of achieving complete blockade of IL-6 signalling versus monotherapy with either mAb alone. This is hardly surprising as these mAbs block different targets, resulting in synergistic inhibition. A limitation, however, is that huge amounts of IL-6 are induced to circulate in the form of IL-6–anti-IL-6 complexes due to the continuous IL-6 production (15). When administered with siltuximab, tocilizumab, despite its 100-fold lower affinity than siltuximab, adds synergistic inhibition by blocking binding of the remaining low level of free IL-6 (not complexed with siltuximab) to IL-6R or sIL-6R. Interestingly, the authors of the meta-analysis showing that therapies directed against IL-6 signalling reduce mortality in patients hospitalised with COVID-19 (16) also concluded that combination treatment strategies may be more effective at inhibiting the inflammatory response than monotherapy.

CRP is an easy marker with which to assess IL-6 bioactivity in clinical practice, with good temporal resolution, as basic research has shown that IL-6 is essential for CRP production by hepatocytes in humans (18, 19). The diffusion of mAbs into the liver is likely higher than in local sites of IL-6 production, such as alveoli in patients with COVID-19. Therefore, CRP production could be fully inhibited while IL-6 bioactivity is only partially inhibited at the site of inflammation. Thus, full inhibition of CRP production is a mandatory but not sufficient requirement for the efficacy of therapies directed against IL-6 signalling.

A limitation of our model is the uncertainty around the diffusion of mAbs into BAF and lymph nodes, particularly when inflammation is present. mAb biodistribution studies have shown a lower concentration of mAbs reported in different tissues [ranging from 0.01% in bronchoalveolar lavage fluid (21) to 14.9% in lung (22) and 8.46% in lymph node (22)] relative to plasma (22). In addition, the mAb concentration in BAF relative to plasma is expected to be very low (0.1–0.3%) according to diffusion data for an anti-respiratory syncytial virus mAb (palivizumab) into bronchoalveolar lavage fluid in animal models (21). It remains to be seen how well siltuximab and tocilizumab can penetrate to have a site-specific effect in humans. Our theoretical model suggests that 10% penetration of these mAbs into BAF should ensure efficient antagonism of IL-6 bioactivity; this percentage penetration of the mAbs could be close to reality.

In conclusion, complete IL-6 antagonism is crucial to controlling disease activity in immune-dysregulated diseases such as COVID-19 and iMCD. Based on our algorithm, this can be achieved with anti-IL-6/anti-IL-6R mAbs, used either in a CRP-driven optimisation strategy or a combination strategy depending on the bioclinical situation and dynamics of the dysregulated inflammation. The real-life feasibility of these theoretically defined approaches needs to be evaluated, in particular the side-effect profile of a combined treatment approach. These strategies should be evaluated in a prospective clinical setting.

## Data availability statement

The raw data supporting the conclusions of this article will be made available by the authors, without undue reservation.

## Author contributions

JR defined the concept of this article. BK first developed the mathematical model that was used in this study by HC and ZL. FR and DF performed the iMCD phase II clinical trial from which data are analysed in this study. JR, BK and ZL drafted the manuscript with input from KK and KL on the analysis and interpretation of the data. All the authors discussed the results and evaluated the manuscript critically for important intellectual content, contributing to revisions at each draft of the manuscript. All authors had access to all of the data, approved the final version and agree to be accountable for all aspects of the work in ensuring that questions related to the accuracy or integrity of any part of the work are appropriately investigated and resolved.

## Funding

EUSA Pharma funded medical writing assistance and provided access to the phase II clinical data of siltuximab for analysis.

## Conflict of interest

JR has consulted for NPO Petrovax Pharm, LEO Pharma and EUSA. FR has received consultant fees from EUSA Pharma, GlaxoSmithKline, Karyopharm, Takeda, Sanofi and the Castleman Disease Collaborative Network and research funding from Janssen Pharmaceuticals and Bristol Myers Squibb. DF has received research funding from EUSA Pharma for the ACCELERATE registry and study drug from Pfizer for a clinical trial of sirolimus. He also holds pending provisional patents for “Methods of treating idiopathic multicentric Castleman disease with JAKI/2 inhibition” and “Discovery and validation of a novel subgroup and therapeutic target in idiopathic multicentric Castleman disease.” KK is an employee of EUSA Pharma.

The remaining authors declare that the research was conducted in the absence of any commercial or financial relationships that could be constructed as a potential conflict of interest.

## Publisher’s note

All claims expressed in this article are solely those of the authors and do not necessarily represent those of their affiliated organizations, or those of the publisher, the editors and the reviewers. Any product that may be evaluated in this article, or claim that may be made by its manufacturer, is not guaranteed or endorsed by the publisher.
