# Supplementary material for: Optimisation of anti-interleukin-6 therapy: Precision medicine through mathematical modelling
Source: Front Immunol. 2022 Jul 19;13:919489. doi: 10.3389/fimmu.2022.919489 (PMC9345304; doi:10.3389/fimmu.2022.919489)
Supplement: Supplementary file 1 [file DataSheet_1.docx]

Supplementary Material

# SUPPLEMENTARY FIGURE LEGENDS

**Supplementary Figure 1** Correlation between IL-6 and CRP levels. As CRP is considered a surrogate measure for IL-6, we performed linear regression analysis to delineate the relationship between IL-6 levels and CRP production using clinical data (1). CRP, C-reactive protein; gp, glycoprotein; IL-6, interleukin-6.

**Supplementary Figure 2** Mathematical modelling demonstrating inhibition of IL-6 activity by an anti-IL-6 mAb, anti-IL-6R mAb and their combination. Outputs of a mathematical model of a situation representative of severe COVID-19, with a cytokine storm and massive IL-6 production (3 ng/ml IL-6 in BAF). Penetration of mAb into BAF was modelled at 10% of the concentration in the plasma. Theoretical curves show the efficacy of siltuximab, tocilizumab and a combination of the two mAbs in reducing the formation of gp130 complexes in BAF (**A**) or CRP (**B**) in the circulation; both gp130 complexes and CRP are surrogate measures for IL-6 bioactivity. Panels show (from left to right) the inhibition of gp130 complex formation (**A**) or CRP (**B**) in response to repeated daily injections of siltuximab, tocilizumab or alternating daily injections of siltuximab and tocilizumab. BAF, bronchoalveolar fluid; CRP, C-reactive protein; gp130, glycoprotein 130; IL-6, interleukin-6; IL-6R, interleukin-6 receptor; mAb, monoclonal antibody.

**Supplementary Figure 3** Mathematical modelling demonstrating inhibition of IL-6 activity by an anti-IL-6 mAb, anti-IL-6R mAb and their combination if the local mAb concentration is equal to the concentration in plasma (100%). Outputs of a mathematical model of a situation representative of iMCD (with persistent high IL-6 secretion, at 1 ng/ml; top panels) or severe COVID-19, with a cytokine storm and massive IL-6 production (3 ng/ml IL-6 in BAF; bottom panels). Penetration of mAb into lymph nodes (iMCD) or BAF (COVID-19) is modelled at 100% of the concentration in plasma. Theoretical curves show the efficacy of siltuximab, tocilizumab, or a combination of the two mAbs in reducing the formation of gp130 complexes in BAF or lymph nodes (**A**) or CRP in the circulation (**B**); both gp130 complexes and CRP are surrogate measures for IL-6 bioactivity. Panels show (from left to right) the inhibition of gp130 complex formation (**A**) or CRP (**B**) in response to repeated daily injections of siltuximab, tocilizumab, or alternating daily injections of tocilizumab and siltuximab. BAF, bronchoalveolar fluid; CRP, C-reactive protein; gp130, glycoprotein 130; IL-6, interleukin-6; IL-6R, interleukin-6 receptor; iMCD, idiopathic multicentric Castleman disease; mAb, monoclonal antibody.

**Supplementary Figure 4** Mathematical modelling demonstrating inhibition of IL-6 activity by an anti-IL-6 mAb, anti-IL-6R mAb and their combination if the local mAb concentration is 1% of that in plasma. Outputs of a mathematical model of a situation representative of iMCD (with persistent high IL-6 secretion, at 1 ng/ml; top panels) or severe COVID-19, with a cytokine storm and massive IL-6 production (3 ng/ml IL-6 in BAF; bottom panels). Penetration of mAb into lymph nodes (iMCD) or BAF (COVID-19) is modelled at 1% of the concentration in plasma. Theoretical curves show the efficacy of siltuximab, tocilizumab or a combination of the two mAbs in reducing the formation of gp130 complexes in BAF or lymph nodes (**A**) or CRP in the circulation (**B**); both gp130 complexes and CRP are surrogate measures for IL-6 bioactivity. Panels show (from left to right) the inhibition of gp130 complex formation (**A**) or CRP (**B**) in response to repeated daily injections of siltuximab, tocilizumab or alternating daily injections of tocilizumab and siltuximab. BAF, bronchoalveolar fluid; CRP, C-reactive protein; gp130, glycoprotein 130; IL-6, interleukin-6; IL-6R, interleukin-6 receptor; iMCD, idiopathic multicentric Castleman disease; mAb, monoclonal antibody.

**Supplementary Figure 5:** Sensitivities of (**A**) IL-6, (**B**) IL-6R and (**C**) CRP for characteristic parameters. Sensitivities are shown for IL-6 (**A**), IL-6R (**B**) and CRP (**C**) with respect to IL-6 clearance, mIL-6/IL-6R Kd, sIL-6/IL-6R Kd, CRP production and sIL-6/IL-6R clearance. CRP, C-reactive protein; IL-6, interleukin-6; IL-6R, interleukin-6 receptor; Kd, dissociation constant; m, membrane; s, soluble.

## Supplementary figure reference

1. Luo P, Liu Y, Qiu L, Liu X, Liu D, Li J. Tocilizumab treatment in COVID-19: a single center experience. J Med Virol (2020) 92:814-8. doi: 10.1002/jmv.25801

# Supplementary Methods

## Additional details on mathematic modelling for blocking IL-6 bioactivity

The model was fitted to experimental data for interleukin (IL)-6, glycoprotein (gp)130 and C-reactive protein (CRP) simultaneously with a proportional error model. This was chosen based on the large order of magnitude variation in the data across the experiments.

y_exp_=y_sim_ + b|_ysim_|error

where y_exp_ is biological species, y is experimental response, y_sim_ is biological species y simulation value, and b is the proportional error parameter. IL-6 receptor (IL-6R) and gp130 in the solution form were tagged “s”; IL-6 and mAb could only be in solution form.

The reaction system was divided into a general plasma compartment and an immobile compartment present at the cellular membrane. One of the simplest approaches to model these biological reactions is to use a “curve fitting” to construct a curve (mathematical model) that has the best fit to series of known data. The algorithm of stiff ode15s solver was used with settings as shown in Supplementary Table 1. A tolerance of 1e–5 was chosen for termination of the data-fitting procedure for the step change in estimated parameters and the function value, and 1e–6 for the first-order optimality. This fitting was performed in an iterative fashion as the model contained numerous parameters to allow efficient simultaneous estimation.

A molar equivalent-order kinetic equation was used for the binding of receptor and monoclonal antibody (mAb) in the solution. The kinetic equation of surface-immobilised ligand and protein was used for interactions on the membrane. Catabolism and appeared clearance were also described by mass/molar action. The degradation rate or clearance rate for non-structural proteins was estimated using the half-life (t_1/2_). For example, if the value for the plasma half-life cycle of soluble (s)IL-6R (IL-6R) was 2 hours, κ would be 0.277. This estimation of degradation rates was found to be acceptable for our model after fitting, and aided in reducing the degrees of freedom in the fitting process. In addition, degradation rates for all biological species on the membrane were estimated to be zero based on previous studies (1,2), showing complete recovery in the hepatocyte compartment (e.g., gp130). In plasma, different cases of steady-state IL-6 and sIL-6R levels were simulated with a constant generation rate. The steady-state level of sIL-6R is 75 ng/ml and IL-6 levels are in the range of 1–10,000 pg/ml. The program performed the simulation for 15 days (360 hours) and the observed biological species had to reach a stable level before applying treatment. A simulation can be reproduced using this software and the above settings.

The “Sensitivity Analysis” task of the SimBiology package was used to estimate parameter sensitivities for IL-6, IL-6R and CRP production, as shown in Supplementary Figure 6. This task uses the complex-step derivative approximation, which is often used for metabolic systems. Full dedimensionalisation of the analysis was used to permit comparisons of parameters with widely different orders of magnitude. Calculation for complex-step derivative sensitivities can be represented by the following equations for each parameter:


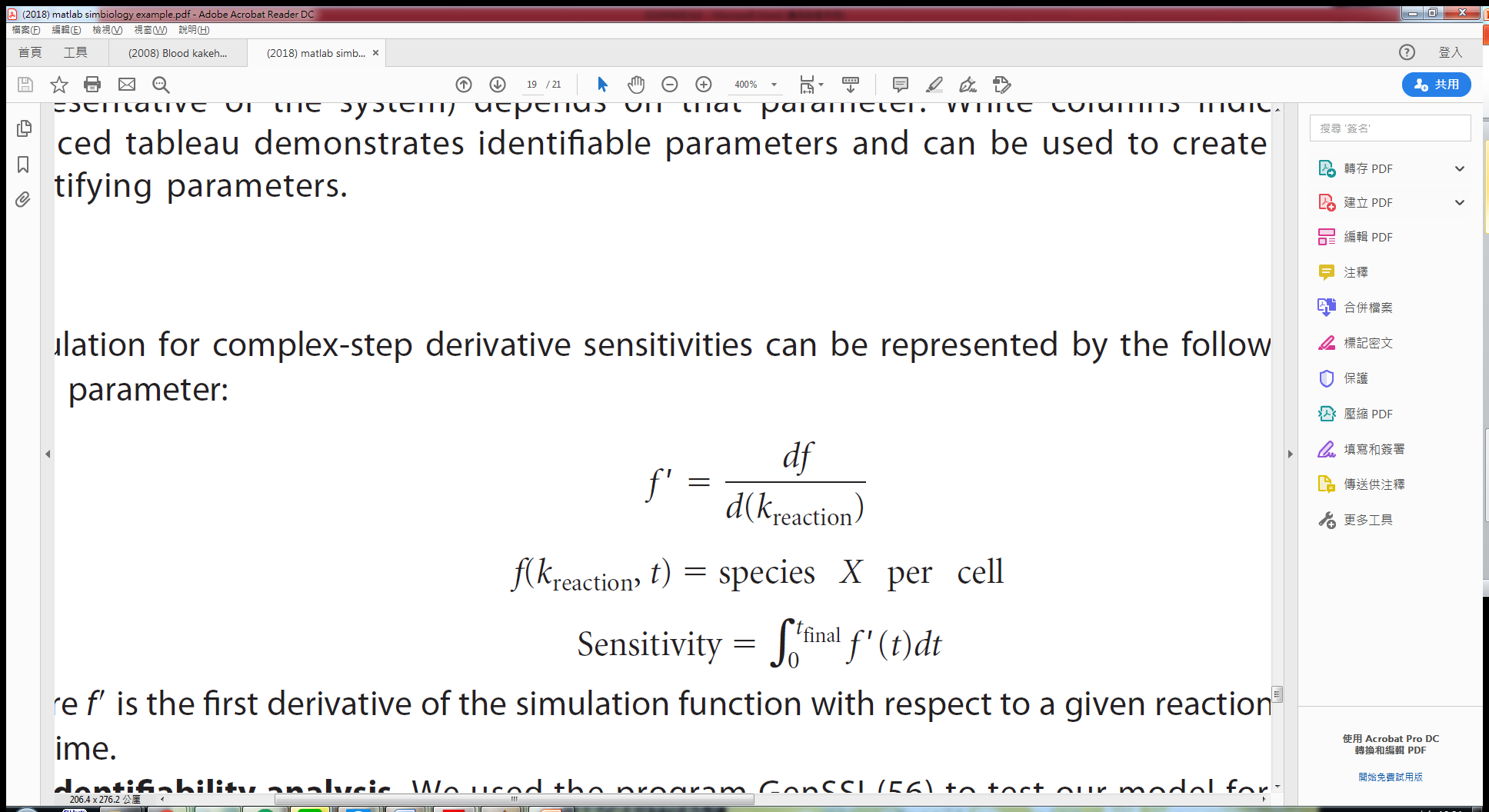


where *f* = is the first derivative of the simulation function with respect to a given reaction rate *k*_reaction_ and *t* is time.

## Modelling inhibition of IL-6/CRP in the presence of siltuximab, tocilizumab or their combination

The diffusion of mAbs into bronchoalveolar lavage fluid is unknown but is expected to be low (~0.1–0.3%) according to the diffusion data for an anti-respiratory syncytial virus mAb into bronchoalveolar lavage fluid in animal models (3). In contrast, the diffusion of mAbs into lymph nodes occurs more freely through the sinusoidal clefts, which allows mAb movement and biodistribution, based on rituximab-mediated B-cell depletion in lymph nodes, and was estimated to be 8.46% of the plasma concentration (4). For this reason, we modelled primarily with local mAb concentrations 10% of those in plasma. To account for the uncertainty (particularly in the scenario of COVID-19), we also modelled various situations using concentrations from 1% to 100% of those in plasma, and present these results as supplementary data.

## References

1. Lu ZY, Brailly H, Rossi JF, Wijdenes J, Bataille R, Klein B. Overall interleukin-6 production exceeds 7 mg/day in multiple myeloma complicated by sepsis. Cytokine (1993) 5:578-82. doi: 10.1016/s1043-4666(05)80007-9

2. Lu ZY, Brailly H, Wijdenes J, Bataille R, Rossi JF, Klein B. Measurement of whole body interleukin-6 (IL-6) production: prediction of the efficacy of anti-IL-6 treatments. Blood (1995) 86:3123-31.

3. Tabrizi M, Bornstein GG, Suria H. Biodistribution mechanisms of therapeutic monoclonal antibodies in health and disease. AAPS J (2010) 12:33-43. doi: 10.1208/s12248-009-9157-5

4. Shah DK, Betts AM. Antibody biodistribution coefficients: inferring tissue distributions of monoclonal antibodies based on the plasma concentrations in several preclinical species and human. Mabs (2013) 5:297-305. doi: 10.4161/mabs.23684

# SUPPLEMENTARY TABLES

**Supplementary Table 1** Reactions and parameters used in mathematic modelling.

| **No.** | **Reaction** | **Setting** | **Biological signification** |
| --- | --- | --- | --- |
| 1 | IL-6 product | 1 pg/ml–10 ng/ml | Increase of IL-6 activity |
| 2 | sIL-6R product | 75 ng/ml | Increase of IL-6 activity |
| 3 | sgp130 product | 800 ng/ml | Decrease of IL-6 activity |
| 4 | gp130 product | 500/cell | – |
| 5 | IL-6R product | 5000/cell | – |
| 6 | CRP product | – | Witness of IL-6 activity |
| 7 | Anti-IL-6 (siltuximab) | 700 mg | Drug dosage |
| 8 | Anti-IL-6R (tocilizumab) | 800 mg | Drug dosage |
| 9 | [IL-6] + [IL-6R] ↔ [IL-6/IL-6R] | Kd=10^–9^ M | Fixation |
| 10 | [IL-6] + [sIL-6R] ↔ [IL-6/sIL-6R] | Kd=10^–9^ M | Fixation |
| 11 | [IL-6/IL-6R] + [gp130] ↔ [IL-6/IL-6R/gp130] | Kd=10^–11^ M | Activation |
| 12 | [IL-6/sIL-6R] + [gp130] ↔ [IL-6/sIL-6R/gp130] | Kd=10^–11^ M | Activation |
| 13 | [IL-6/IL-6R] + [sgp130] ↔ [IL-6/IL-6R/sgp130] | Kd=4×10^–9^ M | Decrease of IL-6 activity |
| 14 | [IL-6/sIL-6R] + [sgp130] ↔ [IL-6/sIL-6R/sgp130] | Kd=4×10^–9^ M | Decrease of IL-6 activity |
| 15 | [anti-IL-6] + [IL-6] ↔ [anti-IL-6/IL-6] | Kd=3.4×10^–11^ M (siltuximab) | Inhibition |
|  |  | Kd=2.5×10^–9^ M (tocilizumab) | Inhibition |
| 16 | [anti-IL-6R] + [IL-6R] ↔ [anti-IL-6R/IL-6R] | – | – |
|  |  | Kd=2.5×10^–9^ M (tocilizumab) | Inhibition |
| 17 | [anti-IL-6R] + [sIL-6R] ↔ [anti-IL-6R/sIL-6R] | – | – |
| 18 | IL-6 clearance | Half-life: 12 min | Decrease of IL-6 activity |
| 19 | sIL-6R clearance | Half-life: 2 h | Decrease of IL-6 activity |
| 20 | sgp130 clearance | – | Increase of IL-6 activity |
| 21 | Clearance of IL-6 sIL-6R complex | – | Decrease of IL-6 activity |
| 22 | Clearance of IL-6 sIL-6R sgp130 complex | Half-life: 19 h | – |
| 23 | CRP clearance | Half-life: 18 h | – |
| 24 | mAb–anti-IL-6 clearance | Half-life: 10 days | Increase of IL-6 activity |
| 25 | mAb–anti-IL-6R clearance | Half-life: 10 days | Increase of IL-6 activity |
| 26 | Clearance of IL-6–anti-IL-6 immune complex | Half-life: 2 h | Immune complex elimination |
| 27 | Clearance of sIL-6R–anti-IL-6R immune complex | 10 days | Immune complex elimination |
| 28 | CRP production | kf=f([IL-6/IL-6R/gp130], [IL-6/sIL-6R/gp130]) | Increase of IL-6 activity |

The mathematical model used the algorithm of stiff ode15s solver with settings shown in this table. Settings were determined using published data (1-6).

CRP, C-reactive protein; gp, glycoprotein; IL-6 interleukin-6; IL-6R, interleukin-6 receptor; mAb, monoclonal antibody; s, soluble.

## References

1. Castell JV, Geiger T, Gross V, Andus T, Walter E, Hirano T, et al. Plasma clearance, organ distribution and target cells of interleukin-6/hepatocyte-stimulating factor in the rat. Eur J Biochem (1998) 177:357-61. doi: 10.1111/j.1432-1033.1988.tb14384.x
2. Lu ZY, Brailly H, Wijdenes J, Bataille R, Rossi JF, Klein B. Measurement of whole body interleukin-6 (IL-6) production: prediction of the efficacy of anti-IL-6 treatments. Blood (1995) 86:3123-31.
3. McArdle PA, McMillan DC, Sattar N, Wallace AM, Underwood MA. The relationship between interleukin-6 and C-reactive protein in patients with benign and malignant prostate disease. Br J Cancer (2004) 91:1755-7. doi: 10.1038/sj.bjc.6602211
4. Puchalski T, Prabhakar U, Jiao Q, Berns B, Davis HM. Pharmacokinetic and pharmacodynamic modeling of an anti-interleukin-6 chimeric monoclonal antibody (siltuximab) in patients with metastatic renal cell carcinoma. Clin Cancer Res (2010) 36:1652-61. doi: 10.1158/1078-0432.CCR-09-2581
5. Paccaly AJ, Kovalenko P, Parrino J, Boyapati A, Xu C, van Hoogstraten H, et al. Pharmacokinetics and pharmacodynamics of subcutaneous sarilumab and intravenous tocilizumab following single-dose administration in patients with active rheumatoid arthritis on stable methotrexate. J Clin Pharmacol (2021) 61:90-104. doi: 10.1002/jcph.1703
6. Mihara M, Kasutani K, Okazaki M, Nakamura A, Kawai S, Sugimoto M, et al. Tocilizumab inhibits signal transduction mediated by both mIL-6R and sIL-6R, but not by the receptors of other members of IL-6 cytokine family. Int Immunopharmacol (2005) 5:1731-40. doi: 10.1016/j.intimp.2005.05.010

**Supplementary Table 2** Approved doses of siltuximab and tocilizumab, and rationale for the doses used in the current mathematical model.

| **Therapy** | **Indication** | **Approved dose^a^** | **Dose used in our mathematical model** | **Rationale** |
| --- | --- | --- | --- | --- |
| Siltuximab (1) | Idiopathic multicentric Castleman disease | 11 mg/kg IV every 3 weeks | 700 mg | Median dose of siltuximab for a person weighing 75–80 kg |
| Tocilizumab (2) | COVID-19 (patients receiving systemic corticosteroids and who require supplemental oxygen or mechanical ventilation) | 8 mg/kg IV single dose | 800 mg | Higher dose of tocilizumab because some patients being treated for COVID-19 have received two injections of 400 mg each |
|  |  | One additional 8 mg/kg IV dose if clinical signs or symptoms worsen or do not improve after the first dose |  |  |
|  | Giant cell arteritis | 162 mg SC weekly in combination with a tapering course of glucocorticoids |  |  |
|  | Cytokine release syndrome | 8 mg/kg IV single dose in patients weighing ≥30 kg |  |  |
|  |  | 12 mg/kg IV single dose in patients weighing <30 kg |  |  |
|  | Systemic juvenile idiopathic arthritis | 162 mg SC weekly in patients aged ≥2 years |  |  |
|  |  | 162 mg SC once every 2 weeks in patients weighing <30 kg |  |  |
|  | Polyarticular juvenile idiopathic arthritis | 162 mg SC once every 2 weeks in patients aged >2 years |  |  |
|  |  | 162 mg SC once every 3 weeks in patients weighing <30 kg |  |  |

^a^Doses of siltuximab and tocilizumab for their approved therapeutic indications are based on the EU Summary of Product Characteristics (1,2) – please refer to full local prescribing information as indications may differ across territories.

IV, intravenous; SC, subcutaneous.

## References

1. European Medicines Agency. SYLVANT 100 mg powder for concentrate for solution for infusion/SYLVANT 400 mg powder for concentrate for solution for infusion. Summary of product characteristics. https://www.ema.europa.eu/en/documents/product-information/sylvant-epar-product-information_en.pdf [Accessed March 2022].
2. European Medicines Agency. RoActemra 20 mg/mL concentrate for solution for infusion. Summary of product characteristics. https://www.ema.europa.eu/en/documents/product-information/roactemra-epar-product-information_en.pdf [Accessed March 2022].

**Supplementary Table 3** Outputs of a mathematical model in situations representative of iMCD with persistent high IL-6 secretion (at 1 ng/ml) and severe COVID-19 with a cytokine storm and massive IL-6 production (3 ng/ml IL-6 in BAF).

| **Scenario** | **Modelled intervention** | **Daily injection schedule** | **Plateau of inhibition (%) at given local mAb concentration** | | | | |
| --- | --- | --- | --- | --- | --- | --- | --- |
|  |  |  | **Local mAb concentration relative to  that in the plasma (%)** | | | | |
|  |  |  | **1** | **10** | **20** | **40** | **100** |
| iMCD | Siltuximab | D0, D1, D2, D3, ---, Dx | 35 | 82 | 90 | 95 | 97 |
|  | Tocilizumab | D0, D1, D2, D3, ---, Dx | 20 | 60 | 75 | 87 | 94 |
|  | Siltuximab + tocilizumab | Siltuximab: D0, D2, ---, Dx ;  Tocilizumab: D1, D3, ---, Dx+1 | 39 | 94 | 98 | 99.5 | 100 |
| COVID-19 | Siltuximab | D0, D1, D2, D3, ---, Dx | 12 | 65 | 85 | 92.2 | 95 |
|  | Tocilizumab | D0, D1, D2, D3, ---, Dx | 9 | 38 | 70 | 82 | 86 |
|  | Siltuximab + tocilizumab | Siltuximab: D0, D2, ---, Dx ;  Tocilizumab: D1, D3, ---, Dx+1 | 17 | 85 | 95 | 99 | 100 |

Efficacy is shown for siltuximab, tocilizumab and a combination of the two mAbs in reducing the formation of gp130 complexes (a surrogate measure for IL-6 bioactivity).

BAF*,* bronchoalveolar fluid; gp130, glycoprotein 130; IL-6, interleukin-6; mAb, monoclonal antibody.
