# Supplementary material for: Optimisation of anti-interleukin-6 therapy: Precision medicine through mathematical modelling
Source: Front Immunol. 2022 Jul 19;13:919489. doi: 10.3389/fimmu.2022.919489 (PMC9345304; doi:10.3389/fimmu.2022.919489)
Supplement: Supplementary file 2 [file Presentation_1.pptx]

## Slide 1
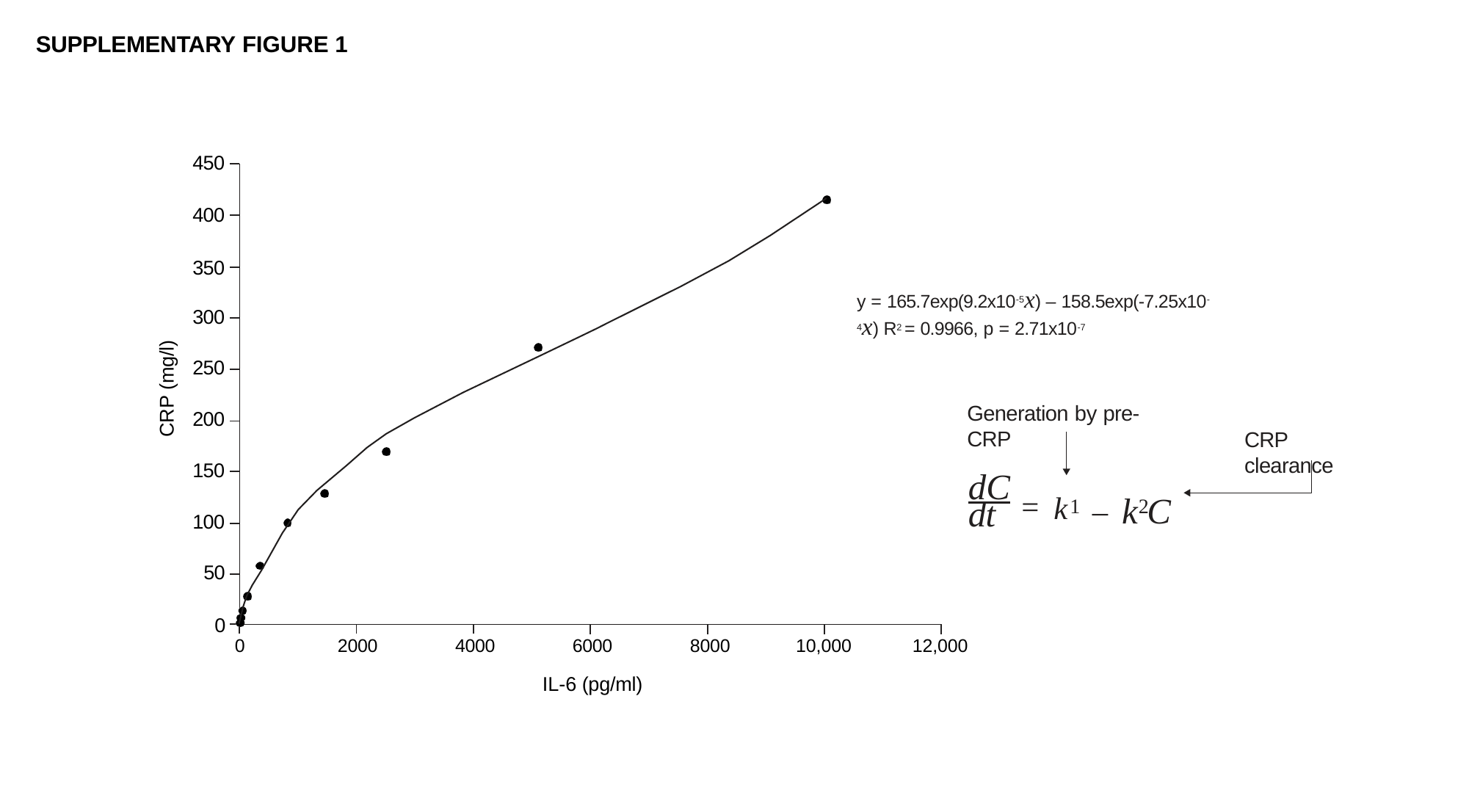

SUPPLEMENTARY FIGURE 1
450
400
350
y = 165.7exp(9.2x10-5x) – 158.5exp(-7.25x10-4x) R2 = 0.9966, p = 2.71x10^-7^
300
CRP (mg/l)
250
Generation by pre-CRP
200
CRP clearance
dC = k
150
– k C
dt
1
2
100
50
0
0
2000
4000
6000
IL-6 (pg/ml)
8000
10,000
12,000

## Slide 2
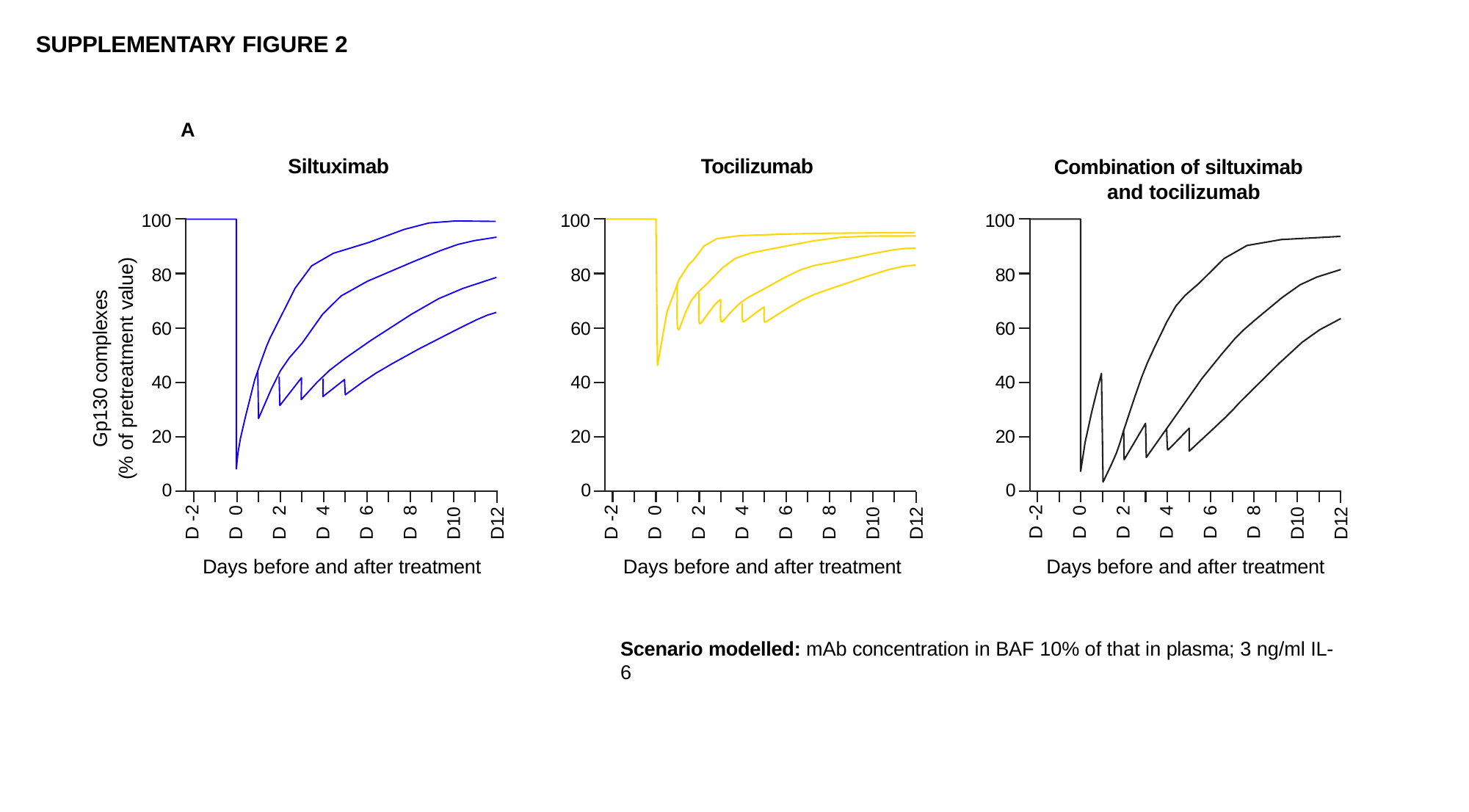

SUPPLEMENTARY FIGURE 2
A
Siltuximab
Tocilizumab
Combination of siltuximab and tocilizumab
100
100
100
Gp130 complexes
(% of pretreatment value)
80
80
80
60
60
60
40
40
40
20
20
20
0
0
0
D10
D12
D10
D12
D10
D12
D -2
0
2
4
6
8
D -2
0
2
4
6
8
D -2
0
2
4
6
8
D
D
D
D
D
D
D
D
D
D
D
D
D
D
D
Days before and after treatment
Days before and after treatment
Days before and after treatment
Scenario modelled: mAb concentration in BAF 10% of that in plasma; 3 ng/ml IL-6

## Slide 3
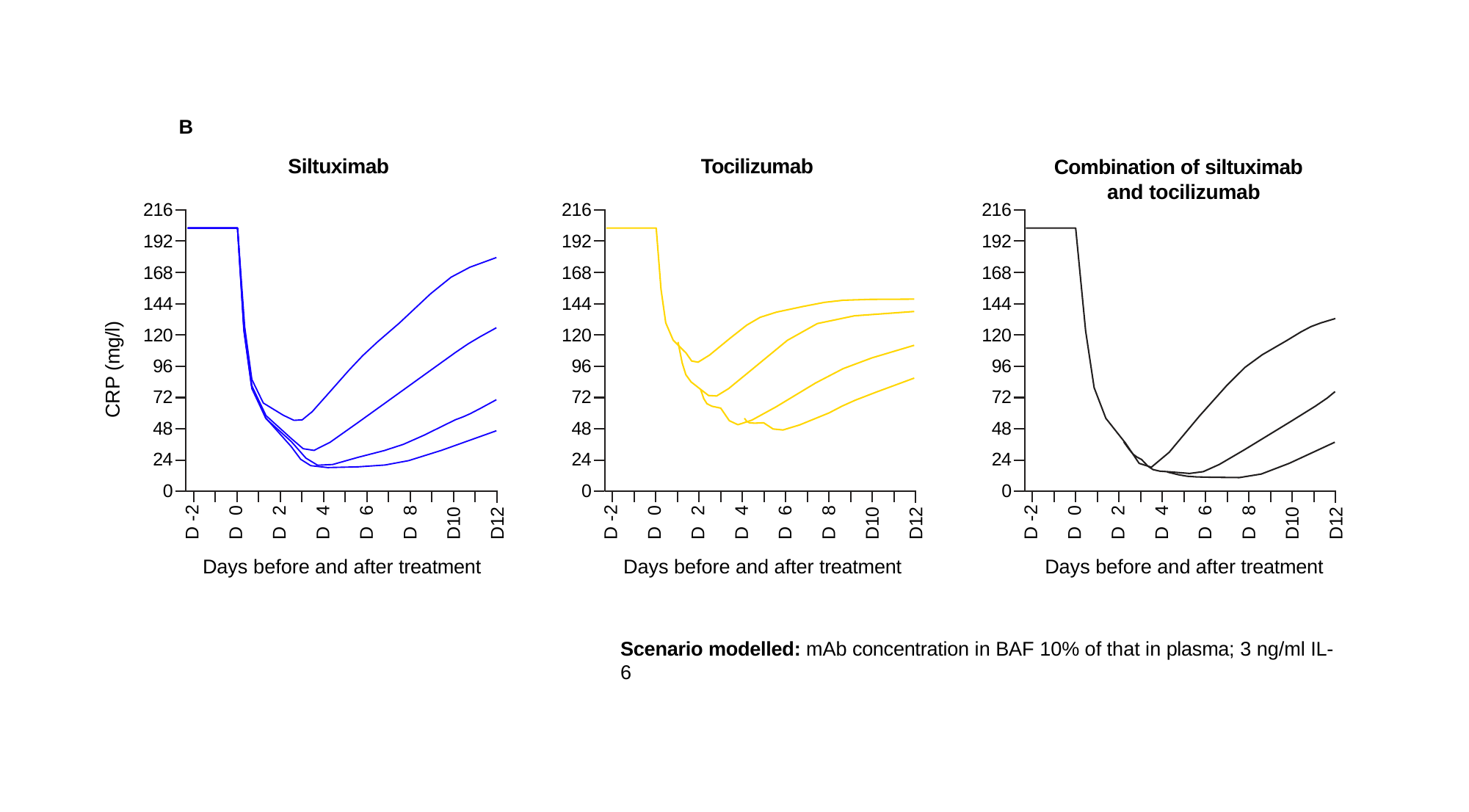

B
Siltuximab
Tocilizumab
Combination of siltuximab and tocilizumab
216
192
168
144
120
96
72
48
24
0
216
192
168
144
120
96
72
48
24
0
216
192
168
144
120
96
72
48
24
0
CRP (mg/l)
D10
D12
D10
D12
D10
D12
D -2
D -2
D -2
0
2
4
6
8
0
2
4
6
8
0
2
4
6
8
D
D
D
D
D
D
D
D
D
D
D
D
D
D
D
Days before and after treatment
Days before and after treatment
Days before and after treatment
Scenario modelled: mAb concentration in BAF 10% of that in plasma; 3 ng/ml IL-6

## Slide 4
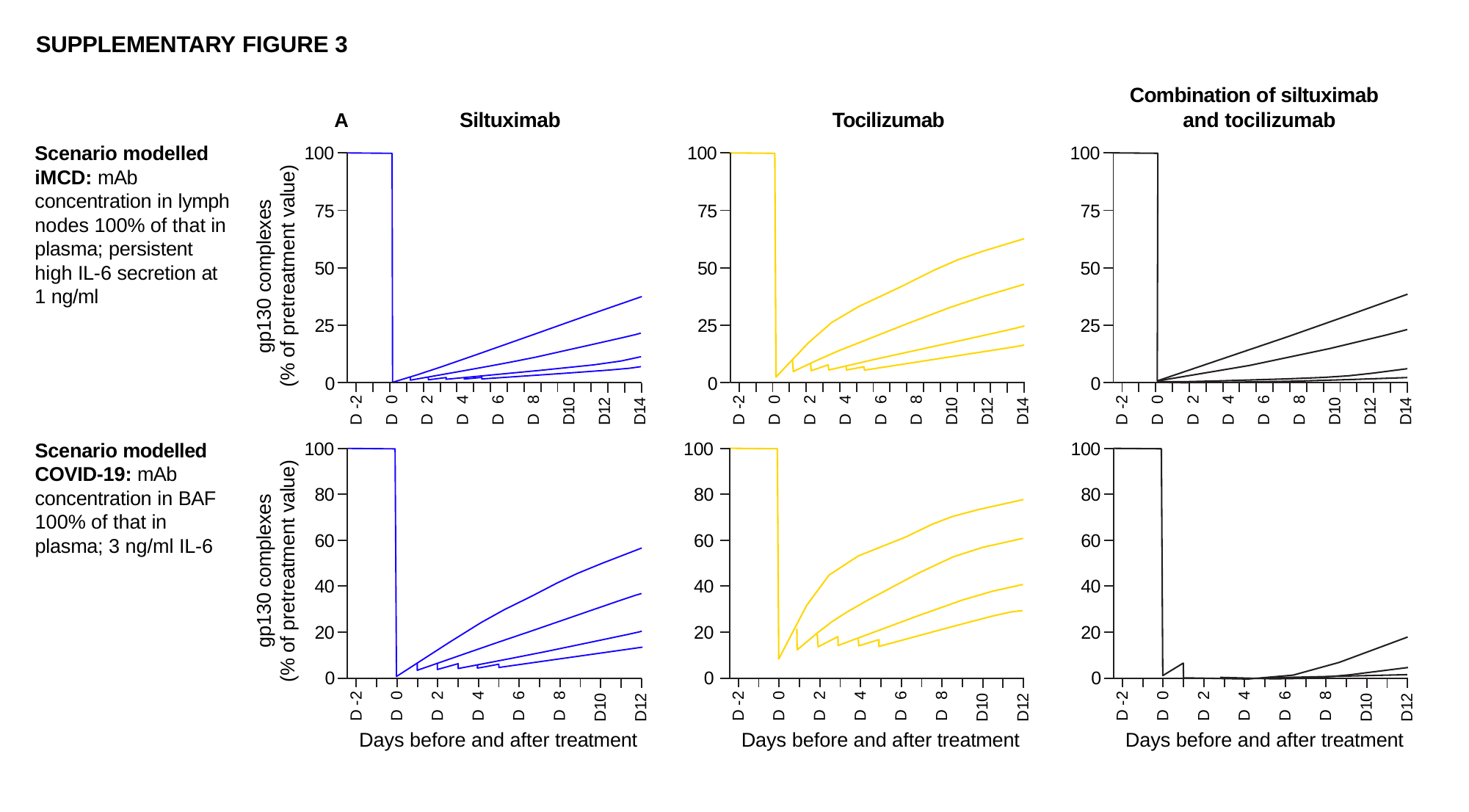

SUPPLEMENTARY FIGURE 3
Combination of siltuximab and tocilizumab
Siltuximab
Tocilizumab
A
Scenario modelled iMCD: mAb concentration in lymph nodes 100% of that in plasma; persistent high IL-6 secretion at 1 ng/ml
100
100
100
gp130 complexes
(% of pretreatment value)
75
75
75
50
50
50
25
25
25
0
0
0
D10
D12
D14
D10
D12
D14
D10
D12
D14
D -2
D -2
D -2
0
2
4
6
8
0
2
4
6
8
0
2
4
6
8
D
D
D
D
D
D
D
D
D
D
D
D
D
D
D
100
100
100
Scenario modelled COVID-19: mAb
concentration in BAF 100% of that in plasma; 3 ng/ml IL-6
gp130 complexes
(% of pretreatment value)
80
80
80
60
60
60
40
40
40
20
20
20
0
0
0
D10
D12
D10
D12
D10
D12
D -2
0
2
4
6
8
D -2
D -2
0
2
4
6
8
0
2
4
6
8
D
D
D
D
D
D
D
D
D
D
D
D
D
D
D
Days before and after treatment
Days before and after treatment
Days before and after treatment

## Slide 5
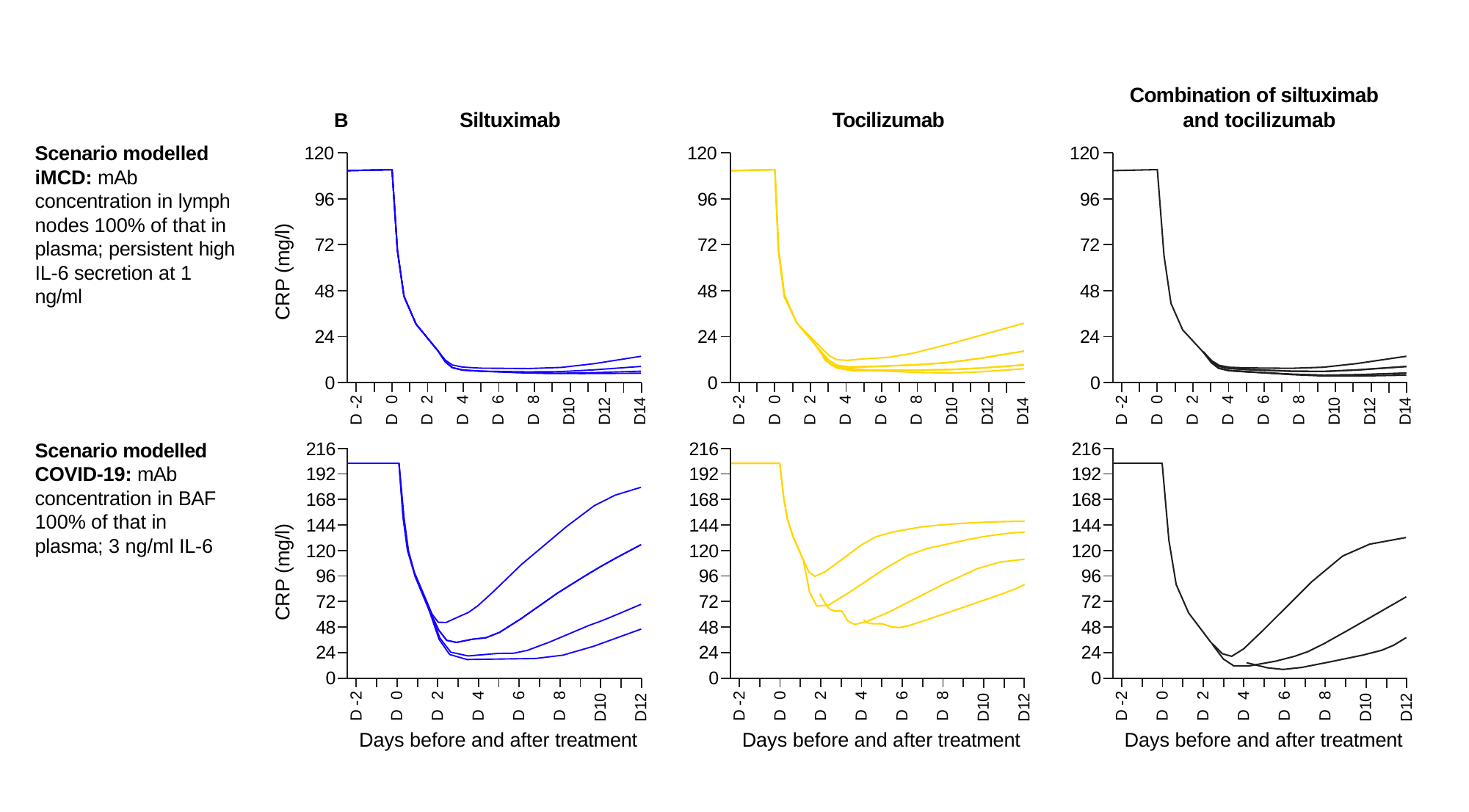

Combination of siltuximab and tocilizumab
Siltuximab
Tocilizumab
B
Scenario modelled iMCD: mAb concentration in lymph nodes 100% of that in plasma; persistent high IL-6 secretion at 1 ng/ml
120
120
120
96
96
96
CRP (mg/l)
72
72
72
48
48
48
24
24
24
0
0
0
D10
D12
D14
D10
D12
D14
D10
D12
D14
D -2
D -2
D -2
0
2
4
6
8
0
2
4
6
8
0
2
4
6
8
D
D
D
D
D
D
D
D
D
D
D
D
D
D
D
216
192
168
144
120
96
72
48
24
0
216
192
168
144
120
96
72
48
24
0
216
192
168
144
120
96
72
48
24
0
Scenario modelled COVID-19: mAb
concentration in BAF 100% of that in plasma; 3 ng/ml IL-6
CRP (mg/l)
D10
D12
D10
D12
D10
D12
D -2
0
2
4
6
8
D -2
D -2
0
2
4
6
8
0
2
4
6
8
D
D
D
D
D
D
D
D
D
D
D
D
D
D
D
Days before and after treatment
Days before and after treatment
Days before and after treatment

## Slide 6
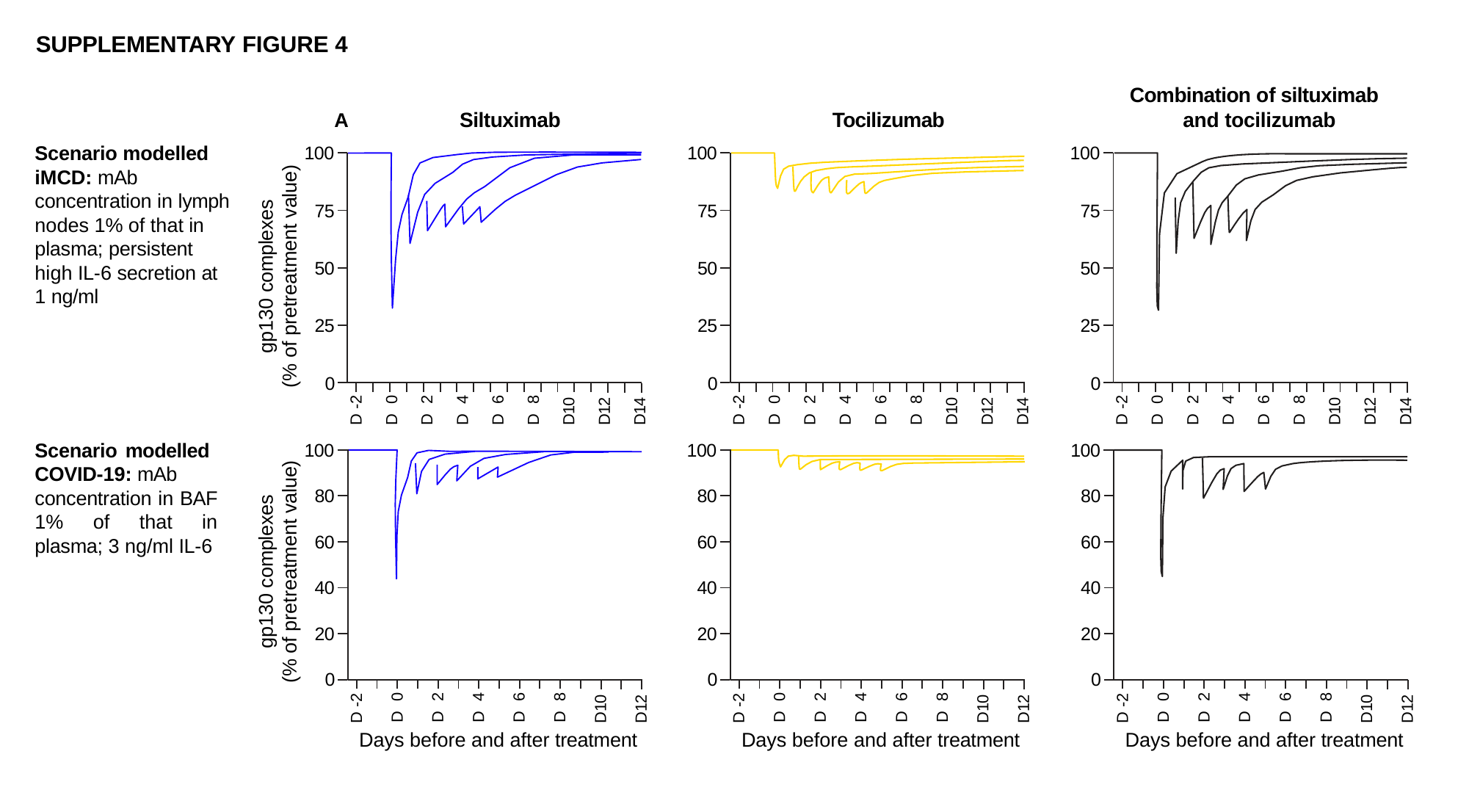

SUPPLEMENTARY FIGURE 4
Combination of siltuximab and tocilizumab
Siltuximab
Tocilizumab
A
Scenario modelled iMCD: mAb concentration in lymph nodes 1% of that in plasma; persistent high IL-6 secretion at 1 ng/ml
100
100
100
gp130 complexes
(% of pretreatment value)
75
75
75
50
50
50
25
25
25
0
0
0
D10
D12
D14
D10
D12
D14
D10
D12
D14
D -2
D -2
D -2
0
2
4
6
8
0
2
4
6
8
0
2
4
6
8
D
D
D
D
D
D
D
D
D
D
D
D
D
D
D
Scenario modelled COVID-19: mAb
concentration in BAF 1% of that in plasma; 3 ng/ml IL-6
100
100
100
gp130 complexes
(% of pretreatment value)
80
80
80
60
60
60
40
40
40
20
20
20
0
0
0
D10
D12
D10
D12
D10
D12
D -2
D -2
D -2
0
2
4
6
8
0
2
4
6
8
0
2
4
6
8
D
D
D
D
D
D
D
D
D
D
D
D
D
D
D
Days before and after treatment
Days before and after treatment
Days before and after treatment

## Slide 7
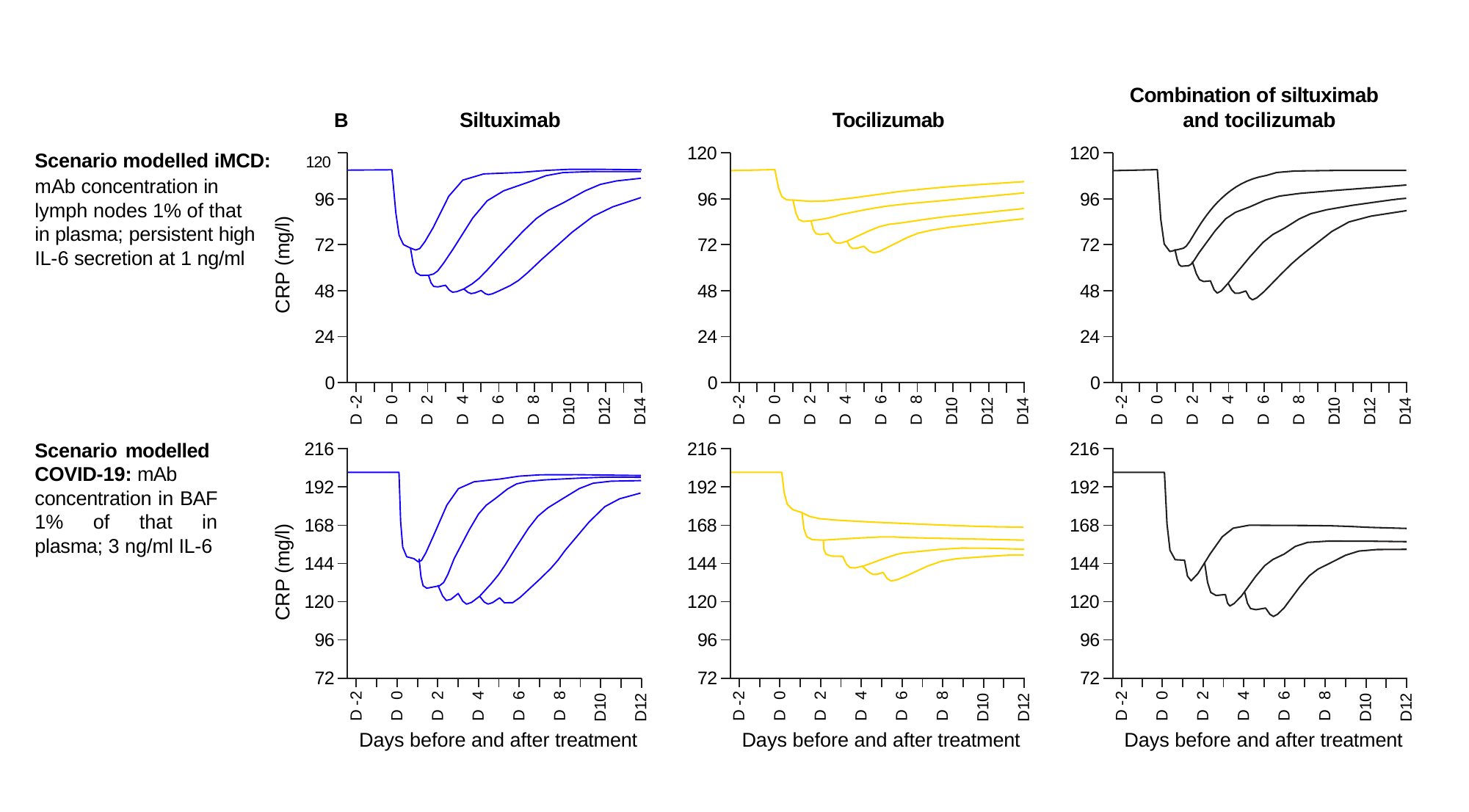

Combination of siltuximab and tocilizumab
Siltuximab
Tocilizumab
B
Scenario modelled iMCD:	120
mAb concentration in lymph nodes 1% of that in plasma; persistent high
IL-6 secretion at 1 ng/ml
120
120
96
96
96
CRP (mg/l)
72
72
72
48
48
48
24
24
24
0
0
0
D10
D12
D14
D10
D12
D14
D10
D12
D14
D -2
D -2
D -2
0
2
4
6
8
0
2
4
6
8
0
2
4
6
8
D
D
D
D
D
D
D
D
D
D
D
D
D
D
D
216
216
216
Scenario modelled COVID-19: mAb
concentration in BAF 1% of that in plasma; 3 ng/ml IL-6
192
192
192
168
168
168
CRP (mg/l)
144
144
144
120
120
120
96
96
96
72
72
72
D10
D12
D10
D12
D10
D12
D -2
D -2
D -2
0
2
4
6
8
0
2
4
6
8
0
2
4
6
8
D
D
D
D
D
D
D
D
D
D
D
D
D
D
D
Days before and after treatment
Days before and after treatment
Days before and after treatment

## Slide 8
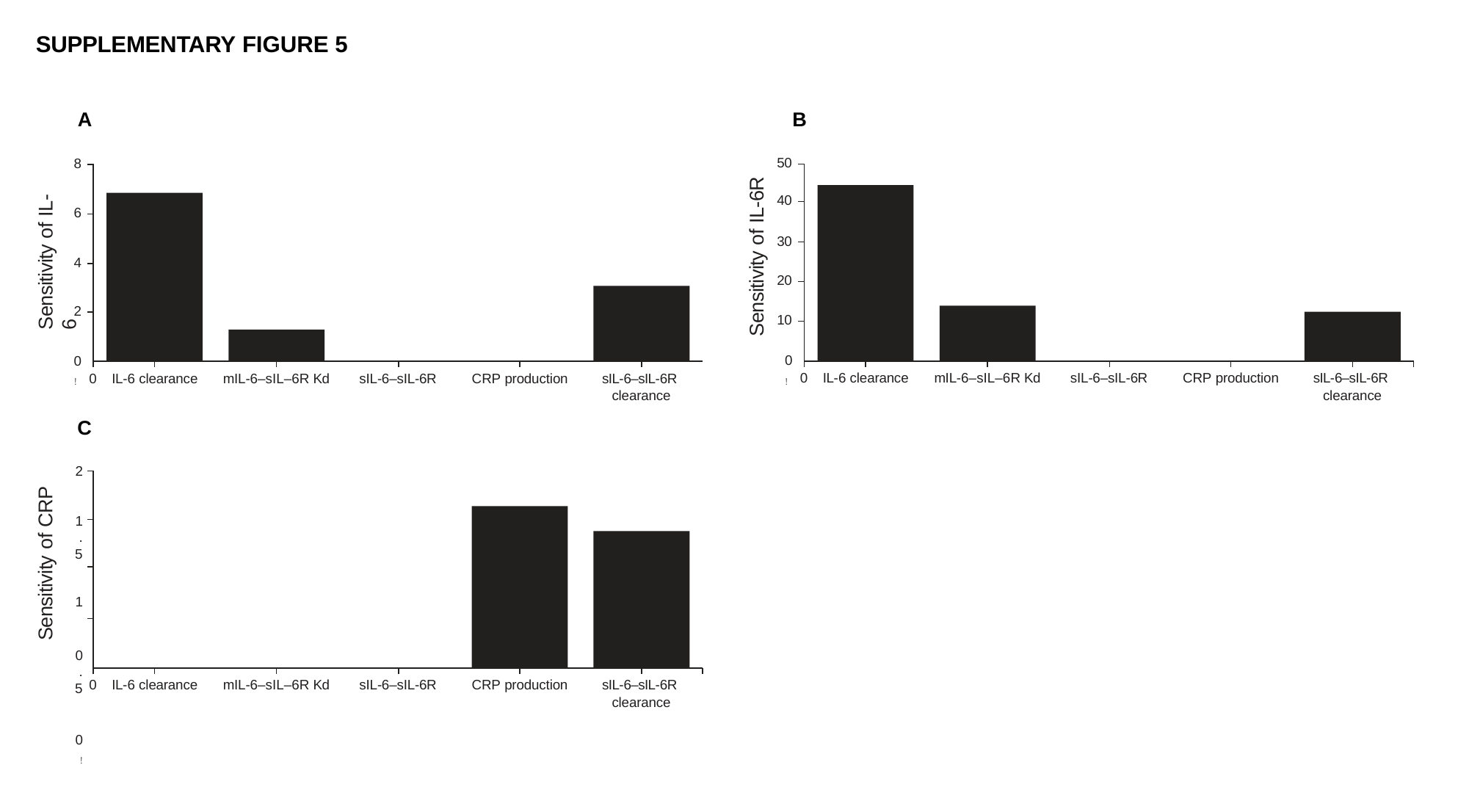

# SUPPLEMENTARY FIGURE 5
A
B
50
8
Sensitivity of IL-6R
Sensitivity of IL-6
40
6
30
4
20
2
10
0!
0!
mIL-6–sIL–6R Kd
sIL-6–sIL-6R
CRP production
sIL-6–sIL-6R
clearance
0 IL-6 clearance
mIL-6–sIL–6R Kd
sIL-6–sIL-6R
CRP production
sIL-6–sIL-6R
clearance
0 IL-6 clearance
C
2
1.5
1
0.5
0!
Sensitivity of CRP
mIL-6–sIL–6R Kd
sIL-6–sIL-6R
CRP production
sIL-6–sIL-6R
clearance
0 IL-6 clearance
